# Sequential LASER ART and CRISPR Treatments Eliminate HIV-1 in a Subset of Infected Humanized Mice

**DOI:** 10.1038/s41467-019-10366-y

**Published:** 2019-07-02

**Authors:** Prasanta K. Dash, Rafal Kaminski, Ramona Bella, Hang Su, Saumi Mathews, Taha M. Ahooyi, Chen Chen, Pietro Mancuso, Rahsan Sariyer, Pasquale Ferrante, Martina Donadoni, Jake A. Robinson, Brady Sillman, Zhiyi Lin, James R. Hilaire, Mary Banoub, Monalisha Elango, Nagsen Gautam, R. Lee Mosley, Larisa Y. Poluektova, JoEllyn McMillan, Aditya N. Bade, Santhi Gorantla, Ilker K. Sariyer, Tricia H. Burdo, Won-Bin Young, Shohreh Amini, Jennifer Gordon, Jeffrey M. Jacobson, Benson Edagwa, Kamel Khalili, Howard E. Gendelman

**Affiliations:** 1 Department of Pharmacology and Experimental Neuroscience, University of Nebraska Medical Center, Omaha, NE 68198-5880 USA; 2 Department of Neuroscience, Lewis Katz School of Medicine at Temple University, Philadelphia, PA 19115 USA; 3 Department of Pharmaceutical Sciences, College of Pharmacy, University of Nebraska Medical Center, Omaha, NE 68198-5880 USA

**Keywords:** CRISPR-Cas9 genome editing, Antivirals, HIV infections

## Abstract

Elimination of HIV-1 requires clearance and removal of integrated proviral DNA from infected cells and tissues. Here, sequential long-acting slow-effective release antiviral therapy (LASER ART) and CRISPR-Cas9 demonstrate viral clearance in latent infectious reservoirs in HIV-1 infected humanized mice. HIV-1 subgenomic DNA fragments, spanning the long terminal repeats and the Gag gene, are excised in vivo, resulting in elimination of integrated proviral DNA; virus is not detected in blood, lymphoid tissue, bone marrow and brain by nested and digital-droplet PCR as well as RNAscope tests. No CRISPR-Cas9 mediated off-target effects are detected. Adoptive transfer of human immunocytes from dual treated, virus-free animals to uninfected humanized mice fails to produce infectious progeny virus. In contrast, HIV-1 is readily detected following sole LASER ART or CRISPR-Cas9 treatment. These data provide proof-of-concept that permanent viral elimination is possible.

## Abstract (web summary)

Here, the authors show that sequential treatment with long-acting slow-effective release ART and AAV9- based delivery of CRISPR-Cas9 results in undetectable levels of virus and integrated DNA in a subset of humanized HIV-1 infected mice. This proof-of-concept study suggests that HIV-1 elimination is possible.

## Introduction

According to UNAIDS, it is estimated that more than 36.7 million people worldwide are infected with the human immunodeficiency virus type one (HIV-1) and >5000 individuals worldwide are newly infected each day. In the clinic, antiretroviral therapy (ART) restricts viral infection by stalling various steps of the viral life cycle. However, ART fails to eliminate integrated copies of HIV-1 proviral DNA from the host genome^1,2^. As such, virus persists in a latent state within infectious reservoirs; and ART cessation readily leads to viral reactivation and disease progression to acquired immunodeficiency syndrome (AIDS)^3^. Thus, a major issue for any HIV-1 curative strategy is the means to eliminate either integrated proviral DNA or the cells that harbor virus without collateral cytotoxic reactions. However, elimination of HIV-1 infection in its infected human host is documented only in two individuals^4,5^. There are several reasons why success has not yet been realized. This includes inadequate therapeutic access to viral reservoirs, rapid spread of infection by continuous sources of virus and susceptible cells and a failure to eliminate residual latent integrated proviral DNA. All single or combination therapeutic approaches preclude HIV-1 cure as viral rebound universally follows ART cessation^6–10^. Yet, another obstacle towards elimination of infection is that viral latency is established after infection onset and precedes peak viremia. This underscores that early intervention with potent antiretroviral medicines may help to further reduce the size of the reservoir and ultimately facilitate viral elimination^11^. Therefore, multimodal robust pharmaceutic strategies are needed for complete elimination of HIV-1 if no viral resurgence after cessation of ART is to be achieved. To address this need and design a suitable therapeutic strategy, our laboratories produce highly hydrophobic lipophilic viral reservoir penetrating antiretroviral prodrugs coined as long-acting slow-effective release ART (LASER ART). LASER ART properties are defined by slow drug dissolution, enhanced lipophilicity, improved bioavailability and limited off-target toxicities, which directly affect the frequency of ART administration from daily to weeks. These reduce disease co-morbidity in small animals and maintains effective antiretroviral drug concentrations in blood and tissue viral reservoirs from days to weeks^12–16^. Macrophages enable uptake of significant amounts of intracellular antiretroviral drug crystals and tightly control ongoing viral replication by the cells’ slow drug release and transfer to adjacent CD4 + T cells during cell-to-cell contact or through direct drug uptake^13,14,16–18^. However, LASER ART alone cannot rid the infected host of latent HIV-1 no matter how successful the drugs may prove to be at restricting viral infection. Thus, in parallel, we develop CRISPR-Cas9 based gene editing technology using AAV_9_ delivery that specifically and efficiently excises fragments of integrated HIV-1 proviral DNA from the host genome^19–24^. We realize that CRISPR-Cas9-based technologies could be most effective in the setting of maximal viral restriction and substantive reductions in the absolute proviral DNA load. Thus, the two approaches are combined to examine whether LASER ART and CRISPR-Cas9 treatments could provide combinatorial benefit for viral elimination. Here we demonstrate elimination of replication competent HIV-1 in an experimental model of human infectious disease. Viral clearance is achieved from HIV-1 infected spleen and lymphoid tissues as well as a broad range of solid organs from documented prior infected humanized mice treated with LASER ART and AAV_9_-CRISPR-Cas9. This is confirmed in those mice using ultrasensitive HIV-1 nucleic acid detection methods by the absence of post-treatment viral rebound; and by the inability to transfer virus from those infected and dual-treated mice to replicate uninfected untreated mice. We conclude that viral elimination by a combination of LASER ART and gene editing strategy is possible.

## Results

### Creation and characterization of HIV-1 infected humanized mice

**Fig. 1:**
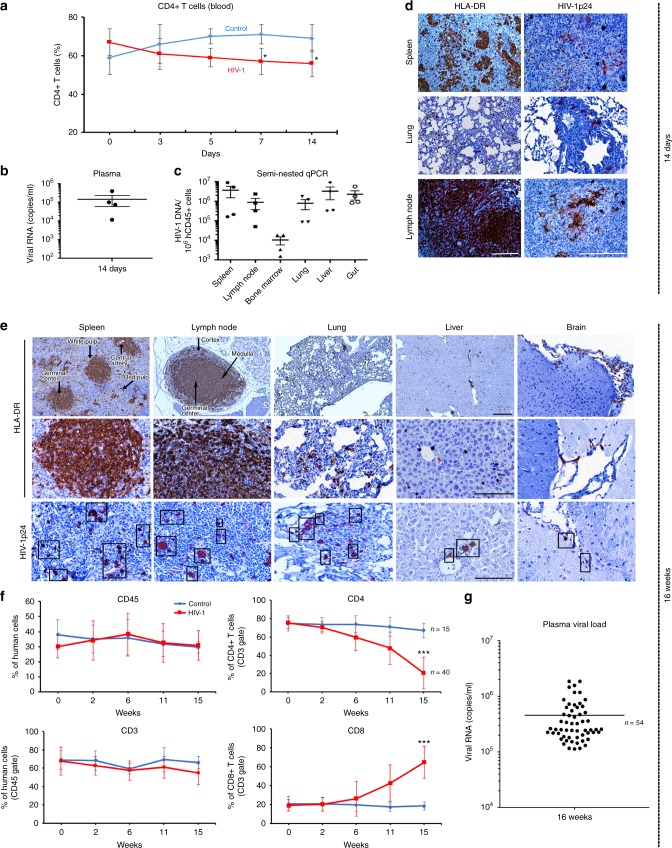
Viral and human immune profiles in a HIV-1 infected humanized mice. **a** Human CD4 + T cells were determined by flow cytometry in blood of humanized mice before infection (time 0) and on days 3, 5, 7, and 14 after HIV-1_ADA_ infection (*n* = 4, red color). Each infected animal received 10^4^ TCID_50_ of titered virus. Uninfected (control, *n* = 3) animals are shown in blue. **b** Viral load measurements in plasma of HIV-1 infected humanized mice at 14 days. **c** HIV-1 DNA was detected by semi-nested real-time qPCR in tissue of infected animals at 14 days after viral infection (*n* = 4). **d** Representative images of human HLA-DR expression in spleen, lung, and lymph nodes, confirms human cell reconstitution in all animals. Replicate sections were stained for HIV-1p24 and show large numbers of infected cells. Scale- bars (10 and 40 μm) **e**–**g** Immune cell profiles and viral load of tissue were evaluated 16 weeks after viral infection**. e** Photomicroscopic images illustrate human cells present in spleens, lymph nodes, lungs, livers and brains of humanized mice. Tissue sections stained with anti- human HLA-DR (upper 2 panels) and HIV-1p24 (bottom panels). **f** Total human CD45 + leukocytes, CD3 + and subpopulations of CD4 + and CD8 T + cells from blood of control (*n* = 15) and HIV-1 infected (*n* = 40) mice. **g** Plasma viral load was consist among the animals in both acute (14 days) and chronic (16 weeks) infectious paradigms, *n* = 54. One-way ANOVA and Bonferroni’s post-hoc tests for multiple comparisons and two-tailed Student’s *t-test* were used for statistical analyses in  a and f. *P < 0.05, ***P < 0.001. Source data are provided as a source data file

With the knowledge that few small animal models of HIV-1 reflect actual viral reservoirs and long-term infections, another system for study is required. This is based both on known species restrictions for HIV-1 infection and long-term establishment of tissue reservoirs of infection. Human hematopoietic stem cells (HSC) reconstituted NOD.Cg-Prkdc^scid^ Il2rgt^m1Wjl^/SzJ (NSG) mice produce human T cells, that are broadly susceptible to HIV-1 infection^23–30^. The model permits evaluation of long-term viral infection in blood and tissues and ART-induced HIV-1 latency. To affirm the model’s relevance for studies of HIV-1 elimination, we undertook a detailed evaluation of each of the human cell-virus model components (Fig. 1). *First*, after irradiation of mice at birth, animals were engrafted with human CD34 + HSC isolated from cord blood by a single intrahepatic injection. The presence of human immunocytes in blood was confirmed by flow cytometry. *Second*, four months after humanization was confirmed animals were infected with HIV-1_ADA_ at 10^4^ tissue culture infection dose_50_ (TCID_50_)/animal and analyzed for acute (14 days) (Fig. 1a–d) and chronic (16 weeks infection) (Fig. 1e–g) paradigm. At sacrifice, human cell reconstitution was confirmed in tissues (spleen, lymph node, liver, lung and brain) by immunohistochemical staining with human HLA-DR antibodies. Anatomical localizations and lymphocyte prominence were confirmed by human cell penetration into the white and red pulp and germinal centers of spleen. Lymph nodes were enriched with human cells with anatomical distinctions in the cortex, medulla and germinal centers. *Third*, productive HIV-1 infection was confirmed by HIV-1p24 staining as shown by large numbers of stained cells. Infection was highest in lymphoid compartments as compared to liver, lung and brain. A significant CD4 + T-cell decline and increased CD8 + T-cell numbers were observed as a consequence of sustained HIV-1 infection. The percentage of human CD4 + T cells in mice was determined in blood by flow cytometry at 2, 6, 11, and 15 weeks and showed decline after infection (Fig. 1f). Plasma viral RNA copies/ml 16 weeks after HIV-1 infection were readily observed (Fig. 1g).

### Generation and pharmacokinetic (PK) testing of LASER ART

We posit that use of CRISPR-Cas9 strategies developed for the elimination of HIV-1 proviral DNA are aided by optimal control of viral replication leading to sustained viral latency (supplementary fig. 1). To this end amongst others, our laboratories have developed LASER ART that facilitates sustained inhibition of viral replication by long-acting hydrophobic lipophilic antiretroviral nanoparticles^11–15^. To accomplish this goal, fatty-acid-modified prodrugs were synthesized as prodrugs for dolutegravir (DTG), lamivudine (3TC) and abacavir (ABC) by esterification with myristic acid. The chemical structures and physicochemical properties were characterized by nuclear magnetic resonance spectroscopy and Fourier-transform infrared spectroscopy, electrospray ionization mass spectrometry and powder X-ray diffraction^11–15^. The LASER ART particles were characterized fully for stability, size, and shape. This included human monocyte-derived macrophage (MDM) nanoparticle drug uptake, release and potency. Data sets were obtained for nanoformulated myristoylated NM (NMDTG), NM3TC and NMABC prodrugs and nanoformulated rilpivirine (NRPV) (Table 1) before being used in the animal studies. These included individual antiretroviral activity for each of the nanoformulations. Moreover, complete  PK profiles were performed for each of the nanoformulated drugs after a single drug nanoformulation injection. These are illustrated with the accompanying dosages administered in BALB/c mice (Table 1). The PK measurements including terminal rate constant (λz) and half-life (t_1/2_), area under the concentration-time curve (AUC), apparent volume of distribution (V_b_/F), total plasma clearance of drug (CL/F), mean resident time (of the unchanged drug in the systemic circulation) (MRT), were outlined in prior works^25–28,12–14,29^. These data sets showed tight control over viral replication, and the short tail of drug removal from blood and tissue affirmed that any lack of viral rebound would accurately reflect residual HIV-1 growth rather than any residual antiretroviral drug present as part of the long-acting regimen.

**Table 1:** Cell and animal PK data sets for the LASER ART nanoformulations

		NMDTG	NM3TC	NMABC	NRPV
Macrophage Uptake, Retention and Antiretroviral Activity	Maximal prodrug uptake (μg/10^6^ cells)	74.3	10.4	11.3	31.6
	Prodrug retention (μg/10^6^ cells)	10.0	ND	5.0	17.9
	Drug Concentration tested (μM)	100	100	100	100
	Multiplicity of infections (MOI)	0.01	0.01	0.01	0.01
	Percent of HIV-1 inhibition (%)	ND	99	99	99
Pharmacokinetics	λz (1/day)	0.0506	0.6584	ND	0.1274
	t_1/2_ (day)	13.77	1.05	ND	5.44
	AUC _last_ (day^a^ ng/ml)	38995.2	1187.0	315.4	13694.9
	AUC _0-∞_ (day^a^ ng/ml)	40727.9	1187.4	1513.8	13706.7
	AUC % Extrapolation	4.34	0.03	79.17	0.086
	V_b_/F (L/kg)	22.1	64.0	ND	25.8
	CL/F (L/day/kg)	1.1	42.1	ND	3.3
	MRT _0-∞_	14.53	2.27	5.53	3.77

Tabular representation of in vitro activity of each of the four nanoformulated long-acting antiretroviral drugs (NMDTG, NM3TC, NMABC, and NRPV). The pharmacokinetic (PK) profile of each of the nanoformulated drugs are illustrated with accompanying doses for mouse testing. The various parameters of PK measurement include terminal rate constant (slowest rate constant), (λz), terminal half-life (t_1/2_), area under the concentration-time curve (AUC), apparent volume of distribution after IM administration (V_b_/F), apparent total plasma or serum clearance of drug after injection (CL/F), mean resident time (of the unchanged drug in the systemic circulation) (MRT). Source data are provided as a source data file.

*HIV-1*_*ADA*_ challenge 10 days after loading

*ND* could not be determined; no significant decline in drug levels from day 1 to day 14 after treatment

^a^Doses: Single IM injection into mice; NMDTG, NMABC and NRPV = 45 mg/kg as DTG, ABC and RPV equivalents; NM3TC = 50 mg/kg as 3TC equivalents

### Editing of viral DNA in ART treated T-cells by CRISPR-Cas9

In earlier studies, we demonstrated editing of HIV-1 proviral DNA by CRISPR-Cas9 in in vitro and ex vivo T cells^18,22^. Here, we adapted a procedure as schematized in supplementary fig 1 and found that at sub-optimum conditions for CRISPR editing of viral DNA, suppression of viral replication by treatment of cells with ART enhances the efficiency of proviral DNA editing by CRISPR. A higher inhibitory effect from LASER-ART compared to those seen in cells treated with conventional ART on HIV-1 expression was observed (Supplementary Fig. 1b and c). Accordingly, cleavage of proviral DNA by (lentiviral), CRISPR-Cas9 was more robust in cells treated with LASER ART than those treated with conventional ART (supplementary fig. 1d and e). The integrity of the editing at the designated sites within the LTR sequences and the specificity of the cleavage were verified by DNA sequences (supplementary fig. 1f). These observations suggest that LASER ART therapy, by keeping the integrated HIV-1 copies to a minimum, improves the ability of CRISPR-Cas9 to edit integrated proviral DNA.

### Viral rebound after LASER ART and AAV_9_-CRISPR-Cas9 treatment of infected humanized mice

**Fig. 2:**
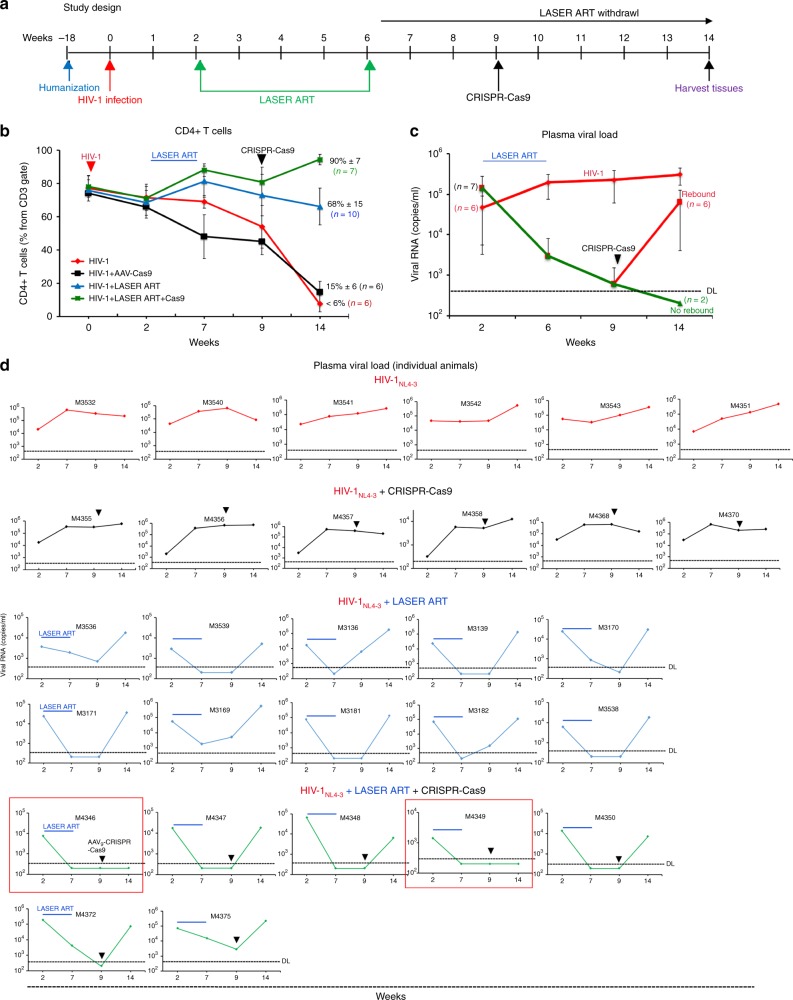
Viral load and CD4 + T cells in HIV-1 infected and treated humanized mice. Mice  were infected with 10^4^ TCID_50_ of HIV-1_NL4–3_ followed by treatments with LASER ART, CRISPR-Cas9 or both. **a** The study scheme shows the times of infection and treatments. After confirmation of viral infection, 29 infected humanized mice were subdivided into four groups. The first group (*n* = 6, red) were left untreated (control), the second group (*n* = 6, black) received a single intravenous (IV) dose of AAV_9_-CRISPR-Cas9 (10^12^ units), nine weeks after viral infection, the third group (*n* = 10, blue) were administered LASER ART (NMDTG and NRPV at 45 mg/kg and NMABC and NM3TC at 40 mg/mg) by intramuscular (IM) injection two weeks after viral infection, the fourth (*n* = 7, green) were given LASER ART (as in group 3) and three weeks after the last LASER ART treatment, a single IV dose of AAV_9_-CRISPR-Cas9 was administered as in group 2. LASER ART treatment was ceased and after an additional five weeks, antiretroviral drug levels were assessed and were at or below the limit of  quantitation < 1 ng/ml (Table 1). **b** Flow cytometry for human CD4 + T cells are shown with increased numbers of CD4 counts in the LASER ART and dual LASER ART and CRISPR-Cas9 groups. **c** Evaluation of plasma viral load indicated that after administration of AAV_9_-CRISPR-Cas9, 2 of 7 mice showed no evidence for viral rebound at 14 weeks. **d** Plasma viral load of individual animals for different treatment groups of humanized mice were assayed at 2, 7, 9, and 14 weeks of HIV-1 infection for HIV-1 RNA. Viral RNA levels were determined by the COBAS Ampliprep-Taqman-48 V2.0 assay with a sensitivity of 200 copies/ml once adjusted to the plasma dilution factor. Viral RNA rebound was observed at the study end in all 10 LASER ART treated animals. This corresponded to eight weeks after therapy interruption. Rebound was also observed at the study end in 5 of 7 dual-treated animals. Virus was not observed in two dual-treated animals (M4346 and M4349) and  are highlighted in the red boxes. Source data are provided as a source data file

**Fig. 3:**
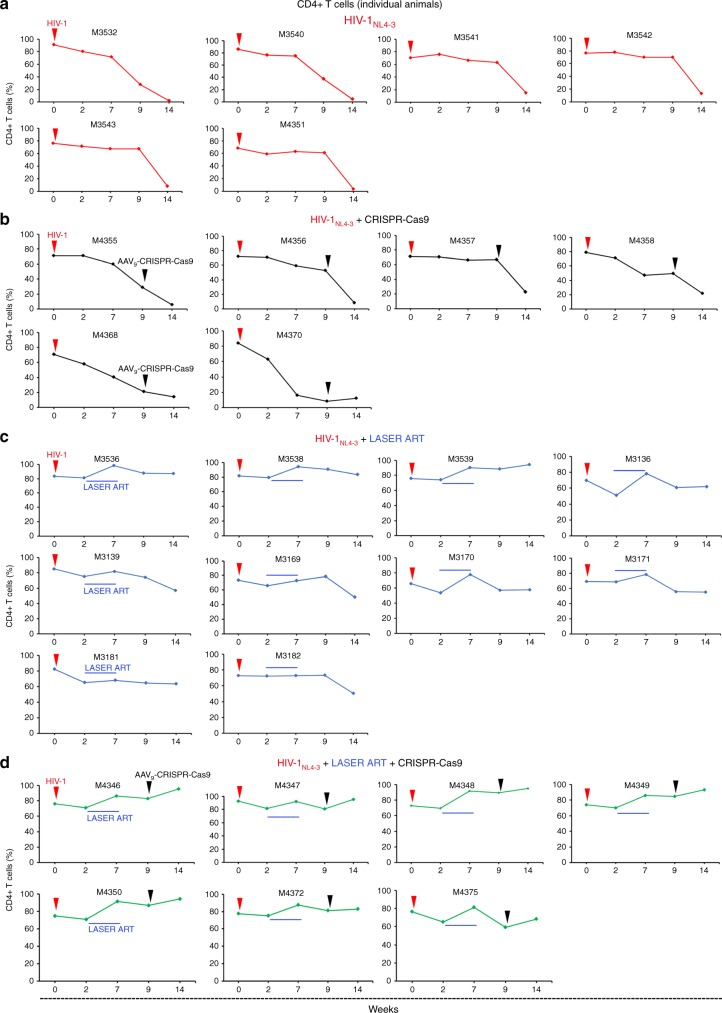
Human CD4 + T cells in HIV-1 infected and treated humanized mice. **a**–**d** Peripheral blood of humanized mice  was assayed before and 2, 7, 9, and 14 weeks after HIV-1_NL4-3_ infection and the presence of human CD4 + cells from CD3 + gated populations were examined. **a** Percentage of human CD4 + T cells followed a decreased pattern in all mice (*n* = 6, red) in the HIV-1 infected group. **b** Percentage of human CD4 + T cells were decreased in all mice (*n* = 6, black) in the HIV-1 infected and AAV_9_-CRISPR-Cas9 group. **c** CD4 + T cell profile of HIV-1 infected and LASER ART animals (*n* = 10, blue) showed a decline in CD4 + T cell numbers two weeks after viral infection. LASER ART was eliminated eight weeks after treatment. **d** CD4 + T cells of HIV-1 infected and LASER ART and AAV_9_-CRISPR-Cas9-treated animals (*n* = 7, green). Decreased CD4 + T cell numbers were seen as early as two weeks after infection. At this time, LASER ART was administered for four weeks followed by AAV_9_-CRISPR-Cas9 given at week 9. The mice were then followed for an additional five weeks. Restoration of CD4 + T cells  was observed in both LASER ART and LASER ART and AAV_9_-CRISPR-Cas9 treatment groups. Source data are provided as a source data file

With the model and therapies in hand, we next evaluated the ability of LASER ART and CRISPR-Cas9 to affect viral rebound after therapeutic interruption  in HIV-1 infected humanized mice (Fig. 2a). In these experiments, HSC reconstituted NSG mice (*n* = 33) were infected with 10^4^ TCID_50_ of HIV-1_NL4-3_ for 2 weeks. Four representative animals were sacrificed at this time point to confirm viral infection establishment from various tissues. At this time, depletion of CD4 + T cells (Fig. 2b) was coincident with plasma viral RNA at a median of 2.2 × 10^5^ copies/ml (Fig. 2c). The remaining 29 HIV-1 infected animals were divided into four groups with four more uninfected untreated animals serving as uninfected controls. The first group (*n* = 6) of mice were left untreated (HIV-1 control), the second group (*n* = 6) received a single intravenous (IV) injection of AAV_9_-CRISPR-Cas9, 10^12^ GC (genome copy) units, with a volume of 50 μl; the third group (*n* = 10) were administered LASER ART that consisted of 45 mg/kg parent drug equivalents of nanoformulated RPV and myristoylated DTG, and 40 mg/kg parent drug equivalents of myristoylated 3TC and ABC nanoparticles by intramuscular (IM) injection. A fourth group (*n* = 7) received LASER ART followed by AAV_9_-CRISPR-Cas9. Eight weeks following the last administration of LASER ART and five weeks after the single AAV_9_-CRISPR-Cas9 treatment animals were observed for evidence of viral rebound (Fig. 2c). In the group that received LASER ART with subsequent AAV_9_-CRISPR-Cas9, viral rebound was not observed in two animals. Examination of the plasma viral load (Fig. 2d) for each individual animal showed drastic decline in the viral copy number to below detectable levels in the group of animals treated with LASER ART. Removal of LASER ART led to rebound in all 10 animals treated with LASER ART alone and in five out of seven animals that received both LASER ART and AAV_9_-CRISPR-Cas9. Repeated search for the viral RNA in the plasma of two animals, M4346 and M4349 (Fig. 2d framed in red), failed to detect evidence of viral presence. In the absence of LASER ART, numbers of CD4^+^ T cells relative to controls were 15 ± 6% and < 6% in groups 2 and 1, respectively (Fig. 2b). The CD4 + T cell profile of each animal is shown (Fig. 3) for all treatment groups. Disease was determined by declining percentages of CD4 + T cells. Results showed a robust restoration of CD4 + T cells in the animals that received LASER ART alone or in combination with AAV_9_-CRISPR-Cas9 as compared to infected controls and AAV_9_-CRISPR-Cas9 alone treated animals (Figs. 2b and 3).

**Fig. 4:**
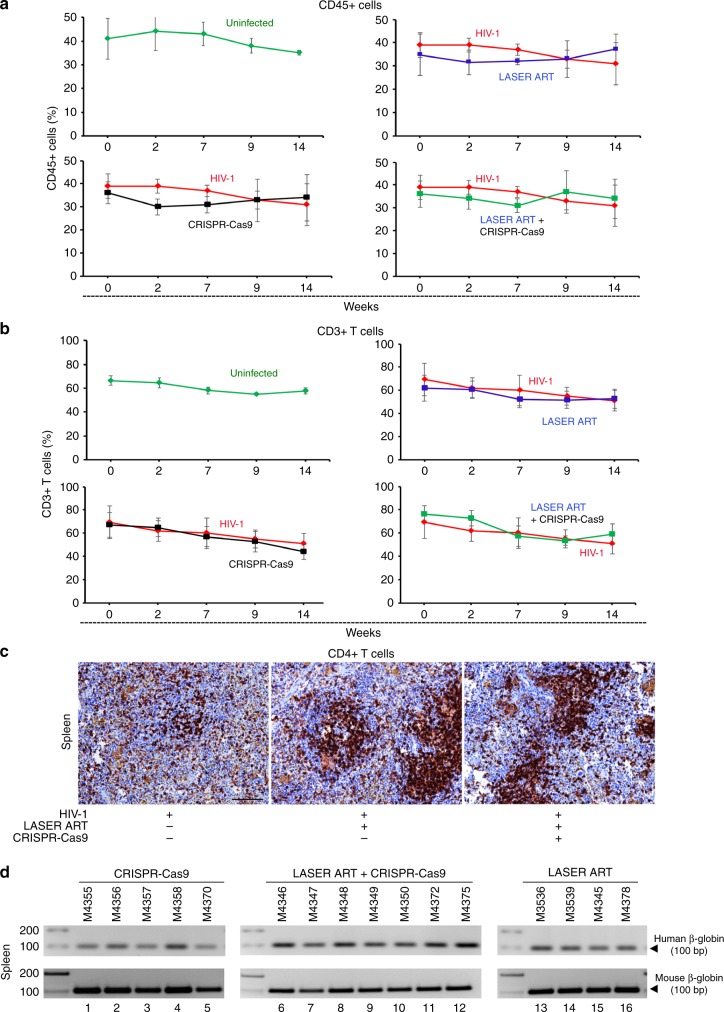
Human leukocytes in blood and spleens of humanized mice. **a**, **b** Peripheral blood of HSC reconstituted mice was assayed before and after 2, 7, 9, and 14 weeks of HIV-**1**_**NL4–3**_ infection for human CD45 + (A) and CD3 + (B) cells. The experiments were performed to assess levels of humanization and percentage of total CD3 + T cells throughout the study. These included uninfected (*n* = 3, green), HIV-1_NL4–3_ infected (*n* = 6, red), HIV-1 and AAV_9_-CRISPR-Cas9-treated (*n* = 6, black), HIV-1 and LASER ART (*n* = 10, blue), and HIV-1 and LASER ART and AAV_9_-CRISPR-Cas9 (*n* = 7, blue/black) treated mice. All are shown from data generated from the experiments outlined in Fig. 3. In the HIV-1 infected mice group, the numbers of CD45 + and CD3 + human cells in blood of mice were comparable to the treatment groups. We did not observe any differences amongst time points when compared to control uninfected and untreated animals. **c** Immunohistochemistry was performed in spleens of HIV-1 infected mice to confirm T cell reconstitution. Here, the spleens of infected animals treated with LASER ART or both LASER ART and CRISPR-Cas9 were examined for the presence and numbers of CD4 + T cells. Significant reductions in CD4 + T cells (brown stained cells)  were seen readily in the HIV-1-infected control mice. These cells were protected in HIV-1 infected animals treated with LASER ART with or without CRISPR-Cas9. Scale bar, 10 μm. **d** Verification of the presence of human cells in the spleens of humanized mice. PCR analysis of genomic DNA isolated from the spleens of humanized mice using primer sets specific to human and mouse beta-globin. Source data are provided as a source data file

Next, we evaluated the number of total human cells (CD45 + ) and T cells (CD3 + ) by flow cytometry and demonstrated sustained human cell numbers in both control (uninfected), infected and treated animals at and beyond four months until the study conclusion (Fig. 4a, b respectively). The presence of human CD4 + cells (Fig. 4c) and HLA-DR in spleen was observed to confirm graft stability. We also observed restoration of CD4 + T cells in spleens of dual-treated animals (Fig. 4c). This was further confirmed by the identification of species-specific DNA sequences in spleens of all animal groups independent of treatments administered (Fig. 4d). Indeed, cell numbers proved constant following all CRISPR-Cas9 and LASER ART interventions.

### HIV-1 elimination in LASER ART and CRISPR-Cas9-treated mice

**Fig. 5:**
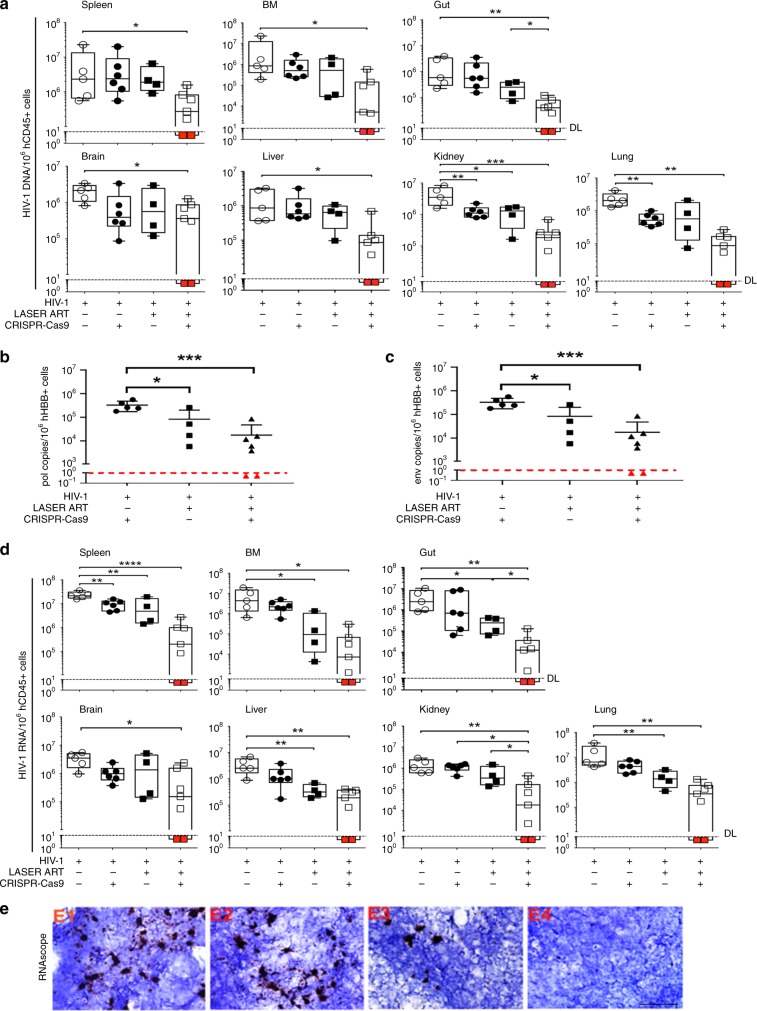
Viral DNA and RNA in HIV-1 infected and treated humanized mouse tissues. **a** HIV-1 DNA and (**d**) HIV-1 RNA analyses using ultrasensitive semi-nested real-time qPCR assays from spleen, bone marrow, gut, brain, liver, kidney, and lung from treatment groups described in Fig. 4a–c. Animal numbers were decreased in one group due to deaths seen through the experimental observation period. The data represent each of the four groups HIV-1 infected (*n* = 5), HIV-1 infected and AAV_9_-CRISPR-Cas9 treated (*n* = 6), HIV-1 infected and LASER ART treated (*n* = 4) and HIV-1 infected LASER ART and AAV_9_-CRISPR-Cas9-treated mice (*n* = 7). The data are expressed as total HIV-1 DNA (**a**) or HIV-1 RNA (**d**) copies/10^6^ human CD45 + cells. Two animals, M4346 and M4349 [shown by the red squares below the dashed lines (detection limit)], with dual treatments, showed sterilization of virus from all tissues analyzed. **b**, **c** Quantitative PCR showed complete elimination of signals corresponding to *pol* (**b**) and *env* (**c**) DNA sequences of HIV-1 in mice M4346 and M4349 (shown as red triangles). One-way ANOVA and Bonferroni’s post-hoc tests for multiple comparisons and two-tailed Student’s *t* test were used for comparisons between two groups for statistical analyses. *P < 0.05, **P *<* 0.01, ***P *<* 0.001, ****P < 0.0001. **e** Representative results from RNAscope assay revealed the detection of single or clusters of brown dots corresponding to HIV-1 RNA in 5 μm-thick spleen sections of infected animals receiving either LASER ART or CRISPR-Cas9 alone, but not both (M4346). *E1*, humanized mice infected with HIV-1 (controls); *E2*, HIV-1 infected animals treated only with CRISPR-Cas9; *E3*, HIV-1 infected LASER ART treated animals demonstrating viral rebound after cessation of therapy; *E4*, infected animals treated with LASER ART followed by CRISPR-Cas9. *E1-E4* are representative tissue sections taken from each of the animal groups. In these assays, we used the antisense V-HIV1-Clade-B targeting 854–8291 bp of HIV-1 as the probe. Scale bar 40μM. Source data are provided as a source data file

Next, we determined viral DNA and RNA levels in tissues (Fig. 5) using ultrasensitive semi-nested real-time qPCR with primers and probes designed for detection of HIV-1 gag. DNA analysis revealed that combination treatment (*n* = 7) was more effective than either LASER ART (*n* = 10) or CRISPR-Cas9 alone (*n* = 6) in DNA copy reductions. The spleen, bone marrow (BM), gut, brain, liver, kidney, and lung of mice M4346 and M4349 showed no rebound. Results from targeted qPCR for DNA sequence detection excluded the presence of DNA corresponding to *pol* and *env* genes in the two, dual-treated and virus eradicated animals (Fig. 5a–c). Similarly, results from the RNA detection assay corroborated these results and showed that combination of LASER ART and CRISPR-Cas9 reduced HIV-1 RNA in select animals with complete absence of viral RNA in M4346 and M4349 (Fig. 5d). The presence of HIV-1 RNA was also examined by RNAscope using 5 μm thick spleen sections from infected animals and antisense probe V-HIV-1 Clade-B designed for targeting base pairs 854–8291 of HIV-1_NL4–3_ (Fig. 5e). Viral DNA and RNA were not detected in plasma or tissues from both mice. Cells and tissues obtained from mouse M4346 contained no viral nucleic acid (Fig. 5e). Further evidence supporting the absence of HIV-1 genomes in animals M4346 and M4349 was provided by digital droplet PCR (ddPCR) (supplementary fig. 2). Verifying prior qPCR results, no viral DNA/RNA (assay’s detection sensitivity of < 2 viral copies) was detected in spleen, bone marrow, and gut of mice M4346 and M4349. The data, taken together, all support the findings of complete HIV-1 elimination. In further cross validation tests, viral rescue assays were performed by co-culturing bone marrow cells and splenocytes of representative animals with phytohemaglutinin/interleukin-2 (PHA/IL-2)-stimulated peripheral blood mononuclear cells (PBMCs). These tests were performed for an additional two weeks. Representative data from these experiments showed that while HIV-1 was rescued from 100% of samples with detectable viral DNA and RNA, no evidence for virus recovery was observed in the samples from the two animals (M4346 and M4349) where HIV-1 DNA and RNA were eliminated despite the presence of high numbers of human cells (supplementary fig. 3).

### On and Off target CRISPR-Cas9 effects

**Fig. 6:**
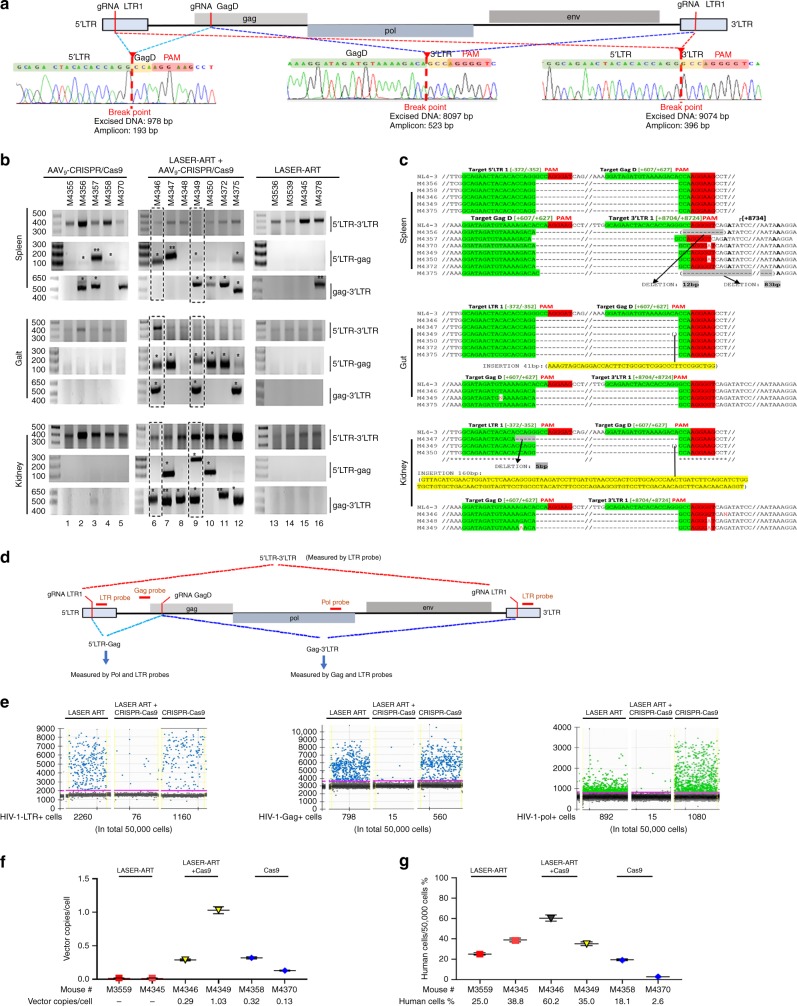
Excision of HIV-1 DNA by CRISPR-Cas9 in HIV-1 infected humanized mice. **a** Schematic illustration of proviral HIV-1_NL4–3_ DNA highlighting the positions of gRNA LTR1 and gRNA GagD target sites, their nucleotide compositions, and the three CRISPR-Cas9 induced break points. **b** Total DNA from spleen, gut, and kidney from three groups of animals used for PCR genotyping with primers sets derived from the 5’LTR, 3’LTR, and the HIV-1 gag gene. Predicted amplicons of 193 bp and 523 bp, which result from the excisions of DNA fragments between 5’LTR to Gag and Gag to 3’LTR, respectively, were selected for DNA sequencing. The fragment of 396 bp represents both populations of full length LTRs, as well as the chimeric of both 5’ and 3’LTR after excision of entire proviral genome. Single asterisks above the bands point to the specificity of fragmental HIV DNA excision by CRISPR-Cas9 as verified by Sanger sequencing (supplementary figs. 3 and 4). The double asterisk depicts non-specific amplicons (supplementary fig. 2). The dashed boxes show the excised HIV-1 amplicons in the two animals with no viral rebound. **c** Representative DNA sequences from each group were aligned to the reference LTR-Gag region of the HIV-1_NL4–3_ sequence. The positions and nucleotide compositions of targets for gRNAs LTR1 and GagD are shown in green, PAM in red, and insertion sequences in yellow. Arrows highlight positions of small and large deletions. **d** Schematic showing locations of each gRNA, the TaqMan probe and the possible excision outcomes. **e** Representative ddPCR data for HIV-1, LTR, Gag, and Pol collected from one HIV-infected humanized mouse of each group treated with LASER ART, LASER ART plus AAV_9_-CRISPR-Cas9 or AAV_9_-CRISPR-Cas9 only (Cas9) are shown. **f** TaqMan probe and primers specific for saCas9, which was delivered by AAV9, were used to determine the AAV transduction efficiency and represented as  AAV vector copies/cell. **g** Total human cell population in samples was measured using TaqMan probe and primers specific for human beta-actin. Source data are provided as a source data file

We next evaluated on and off target CRISPR-Cas9 effects in infected and treated animals. In these experiments, gel electrophoresis was performed in PCR-amplified DNA fragments from infected and treated animals using pairs of primers designed to detect cleavage (Fig. 6). As expected, excision of viral DNA fragments was readily observed from spleen, gut, and kidney samples of animals treated with LASER ART and CRISPR-Cas9 (Fig. 6b). Excision of the predicted fragment in lung, liver, and brain amongst other tissues was also observed (supplementary fig. 4). The excision type differed in the various tissues amongst animals. The integrity and precision of the HIV-1 DNA excision by CRISPR-Cas9 were verified by sequencing (Fig. 6c, and supplementary figs. 5–7). In mice that received CRISPR-Cas9 without LASER ART, fragmental deletion was detected. Several other DNA fragments in tissues from animals that received LASER ART alone were amplified, but after sequencing were found unrelated to HIV or CRISPR-Cas9 editing. This observation likely represented replication defective HIV-1 (highlighted by double asterisks, Fig. 6b). The efficiency of the proviral DNA excision by CRISPR-Cas9 in the spleens  of two infected humanized mice from the CRISPR-Cas9 and LASER ART group (animals where no rebound was observed) was determined by ddPCR. Excision efficiency was estimated to be 80% in both 5′-LTR-Gag and Gag to 3’-LTR in mouse M4349. Transduction efficiency was determined by ddPCR and ranged from 0.12–1.03 AAV vector copies/cell (Fig. 6d–f). Amplification of the DNA fragments corresponding to the control housekeeping actin gene in tissues and expression of gRNAs and Cas9 are shown in supplementary fig 8.

Clustering analysis revealed similar excision patterns with high efficiency across the tissues in animals that received dual treatments compared to those that received CRISPR-Cas9 alone (supplementary fig. 9). However, results from sequencing of several selected sites with high scores of specificities and/or their locations in the exons ruled out off-target effects (supplementary figs. 10 and 11, and supplementary table 3). Further, results from whole genome deep sequencing of DNA from spleens of four treated animals, including two animals that showed no rebound after dual treatment and one from each of the two single treatment groups was performed and then confirmed by bioinformatics analyses. No detectable off target effects on more than one hundred predicted sites that were seen can be attributed to CRISPR. This was done by identifying somatic genomic alterations including structural variants (SVs), single nucleotide polymorphisms (SNPs), copy number variations (CNVs) and small insertion and deletion (InDel) mutations under different treatments and comparing  these with all potential off-targets (supplementary Tables 1–5 and figs. 10–13).

### Validation for LASER ART and CRISPR-Cas9 HIV-1 therapeutic elimination

**Fig. 7:**
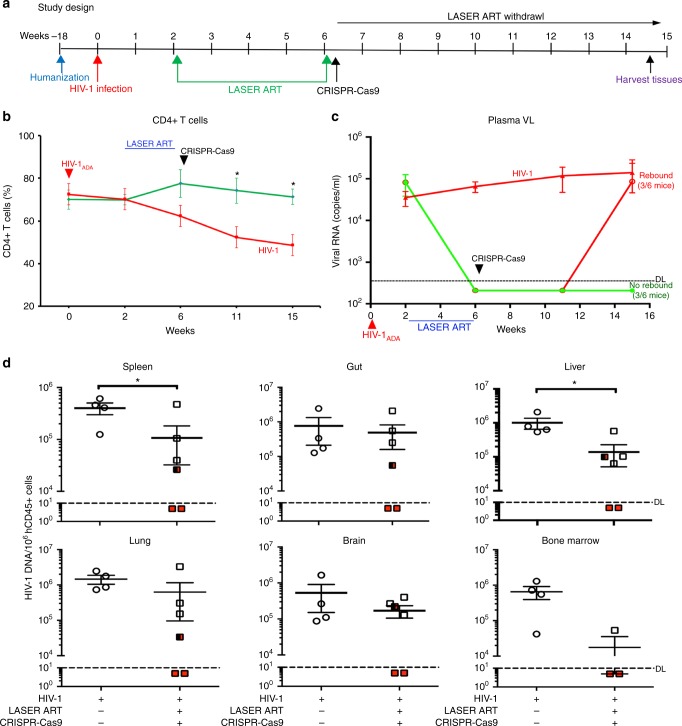
Viral elimination in a subset of HIV-1 infected and treated humanized mice. Dual LASER ART and CRISPR-Cas9 treatment resulted in viral elimination in up to a third of HIV-1_ADA_ infected animals. Validation was made by state of the art viral, immune, and excision detection systems. **a** The timeline of the experiment showing the temporal administration of LASER ART and CRISPR-Cas9 treatments, and animal sacrifice. **b** The percentage of human CD4 + T cells and (**c**) viral loads measured in the HIV-1 infected (*n* = 4, red). Dual treated animals (*n* = 6, green) that showed no (*n* = 3, green) or viral rebound (*n* = 3, green to red) in plasma. **d** HIV-1 DNA analysis was performed using ultrasensitive semi-nested real-time qPCR assays from spleen, gut, liver, lung, brain and bone marrow from infected (*n* = 4, open circles) and infected and dual-treated mice (*n* = 6, squares). Whole bone marrowcells isolated from three animals (out of 6) from the dual-treated rebound group were used for adoptive transfer, Therefore, the data represent *n* = 3 for real-time-PCR assay for the dual-treated animals in the bone marrow adoptive transfer studies. The data are expressed as total HIV-1 DNA copies/10^6^ human CD45 + cells. Two animals, M3319 and M3336 (illustrated by the red squares) were below the dashed lines for virus detection as measured by plasma VL. These animals had no detectable viral DNA after dual treatments demonstrating viral sterilization from all analyzed tissues. A single animal (M3324), as illustrated by a half-red-black designation, had an undetectable VL in plasma, but viral DNA was amplified in all the tissues analyzed. Source data are provided as a source data file

**Fig. 8:**
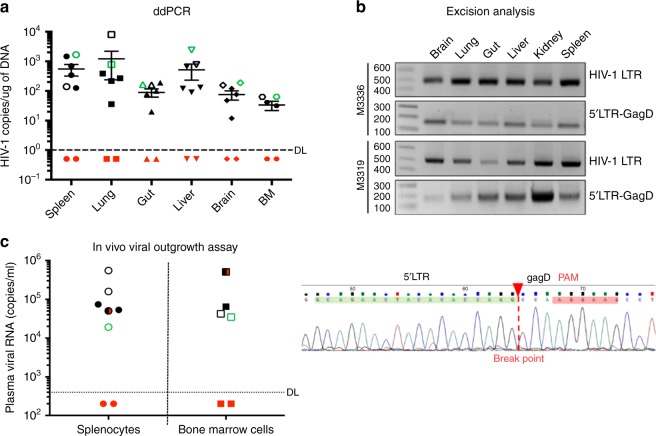
Confirmation of viral elimination in HIV-1 infected and treated mice tissues. **a** Ultrasensitive ddPCR, with sensitivity of detecting 1–2 viral copies, was used in cross validation tests for viral DNA detection and performed in tissues of HIV-1_ADA_-infected and infected/dual-treated animals. As a positive control, one animal each from the HIV-1 infected (open black structure) and HIV-1 and LASER ART (open green structure) groups are illustrated. These were placed together with the six infected animals from the dual treatment group illustrated as closed structures (either black or red). Dashed line represents the limit of detection. Results are shown as the mean ± SEM (BM: bone marrow). **b** Agarose gel analyses of the PCR assay of DNA from various tissues of two animals with no rebound shows the presence of segments of HIV-1 LTR DNA and detection of a 193 bp amplicon, indicative of excision of a DNA fragment between the LTR and the *gag* gene (top). The histogram illustrates representative results from sequencing of the  193 bp fragment highlighting the position of the 5’ LTR breakpoint, and Gag and PAM trinucleotide on the GagD RNA. **c** An in vivo viral outgrowth assay was performed by adoptive transfer of splenocytes and bone marrow cells from infected and virus eradicated LASER ART + CRISPR Cas9-treated mice to uninfected recipient CD34 + NSG-hu mice. These animals failed to show viral recovery after one month of examination by plasma viral RNA measurements. Confirmation assays were performed as positive controls: two animals from an HIV-1 infected  group (open black circles for spleen and boxes for bone marrow) and an animal from a LASER ART treatment group are shown as open green circles (spleen) and box (bone marrow). All controls readily recovered virus. Five animals from the dual treatment group are illustrated as closed circles (spleen) and boxes (bone marrow). Virus was not detected in plasma from animals injected with splenocytes and bone marrow cells isolated from 2 dual-treated animals (M3319 and M3336, red circles and boxes) and used as the definition of viral eradication. Source data are provided as a source data file

To validate eradication of HIV-1 infection, we performed replicate experiments in a separate set of HSC-reconstituted NSG mice infected with a second macrophage tropic viral strain (HIV-1_ADA_) (Fig. 7a). Animals were infected with 10^4^ TCID_50_ of HIV-1_ADA_ for two-weeks, at which time depletion of CD4 + T cells was observed and viral RNA copies in plasma were recorded at a median level of 8.4 × 10^4^ copies/ml (Fig. 7b–c). Semi-nested real-time qPCR of HIV-1 nucleic acid confirmed tissue viral infection from three representative animals. Infected animals were divided into three groups, a no treatment infected group (*n* = 4); those that received LASER ART (combinations of DTG, RPV, 3TC, and ABC) with (*n* = 6) or without CRISPR-Cas9 (*n* = 7). Animals were observed for ten weeks after cessation of LASER ART for viral rebound. Continuous viral replication was readily observed in untreated animals and those treated with only LASER ART. Notably, three of six LASER ART and CRISPR-Cas9-dual-treated animals had no demonstrable viral rebound (Fig. 7c). In these mice, protection of CD4^+^ T cell counts (71.3 ± 3.5%) was observed compared to infected controls (48.3 ± 4.5%) (Fig. 7b). The CD4 + T cell and viral load profiles for each of the individual animals are shown (supplementary figs. 14–15). As in the first experiment, detection of human DNA sequences in spleen confirmed uniform presence of human cells in lymphoid tissues of all humanized mice regardless of treatment. Moreover, no evidence of viral gene expression was found in another two animals (M3319 and M3336) employing qPCR tests for DNA sequence detection (Fig. 7d). HIV-1 genome amplification was also absent in these two mice and were confirmed by the ddPCR (Fig. 8a) and RNAscope tests (supplementary fig. 16). One animal out of three that had no demonstrable rebound in plasma showed detectable HIV-1 DNA in tissues (split red-black structure, Fig. 7d), confirming the important role that tissue reservoirs play during HIV-1 infection. Viral DNA by PCR, gel electrophoresis, and Sanger sequencing verified the ability of CRISPR-Cas9 to excise the target DNA fragment positioned between the LTR and GagD (Fig. 8b and supplementary fig. 17).

Finally, splenocytes and bone marrow cells were isolated from HIV-1 infected mice with or without prior LASER ART and/or CRISPR-Cas9 treatments at the study end. These cells were used in adoptive transfer studies performed in uninfected drug naive humanized mice to examine the potential rebound from latent reservoirs not detected by standard ddPCR and nested PCR. In addition, as positive controls, two animals from the HIV-1 infected group, one from the LASER ART alone treatment group and five animals from the dual treatment group were used as controls for adoptive transfers (Fig. 8c and supplementary fig. 18). Recipient mice were sacrificed after 30 days and analyzed for plasma viral RNA. Virus was not detected in plasma from animals that received splenocytes or bone marrow cells isolated from sequentially LASER ART CRISPR Cas9-treated animals (M3319 and M3336). In contrast, virus was readily identified in all virus infected or virus infected and LASER ART treated animals. One animal each from the HIV-1- and dual-treated bone marrow injected group died prior to the assay performance and as such these data were not included. In conclusion, the in vivo viral outgrowth assays performed from virus eliminated dual-treated mice to new uninfected recipient humanized mice failed to demonstrate viral recovery after one month following adoptive transfers (Fig. 8c). Also, no histopathological evidence for cytotoxicities were detected in any of the animals by conventional microscopic test evaluations of liver (supplementary fig. 19).

To provide an additional level of confirmation to these observations, a third series of studies using replicate procedures were performed. Here, we confirmed the ability of LASER ART and CRISPR-Cas9 to eliminate viral rebound in a new cohort of CD34 + HSC-reconstituted animals infected with HIV-1_ADA_. The outcome of the virological assays showed no evidence of the presence of replication competent virus in four out of ten animals tested after removal of LASER ART in the dual therapy group (supplementary Table 6). All HIV-1 animals that received no or single treatments showed viral rebound after treatment was stopped. Notably, in all three experiments and in all treatment groups, no demonstrable changes in the animals’ well-being or histopathology were observed.

Altogether, the results from three independent sets of studies revealed that a third or more of the animals that received sequential LASER ART and CRISPR-Cas9 therapy became virus free (supplementary Table 6). Most likely, successful outcomes for virus elimination in these animals reflect a combination of factors that include viral set points, extent of a previously established tissue viral reservoir, efficient intracellular and tissue delivery of LASER ART, transduction efficiency and excision therapy at sites of viral growth. Future work will begin to dissect each and all of these factors pointing to viral elimination pathways.

**Fig. 9:**
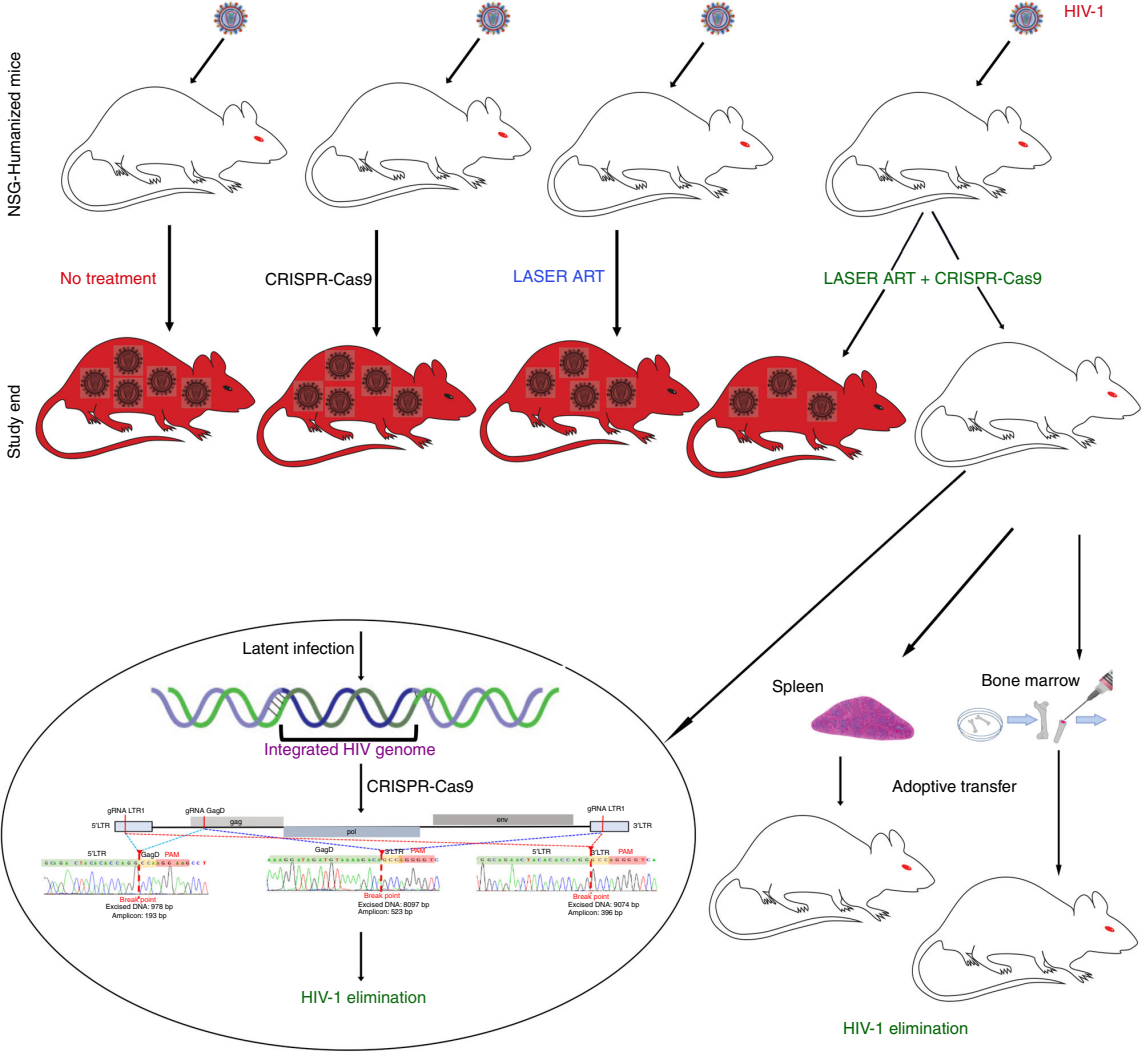
A working model for HIV-1 elimination. A cartoon illustration of the viral elimination strategy is shown for single LASER ART, AAV_9_-CRISPR-Cas9 injection groups and dual treatment groups. We highlight the observed restriction of viral infection by LASER ART and an inability to achieve elimination of virus by AAV_9_-CRISPR-Cas9 treatment alone. However, the sequential administration of LASER ART and AAV_9_-CRISPR-Cas9 can achieve viral elimination in a subset of animals. Why specific animals are cured of infection while others are not is incompletely understood but tied, in measure, to viral set points

LASER ART was administered then removed with consequent CRISPR-Cas9 excision. Plasma drug levels were monitored until they reached levels that were at or below the limit of detection. In these three independent experiments, one with HIV-1_NL4–3_ and the other two with HIV-1_ADA_ infection of humanized mice, single treatments with LASER ART or AAV_9_-CRISPR-Cas9 resulted in viral rebound in 100% of treated infected animals. In dual LASER ART and AAV_9_-CRISPR-Cas9-treated mice, virus was eliminated from cell and tissue reservoirs in up to a third of infected animals as illustrated (Fig. 9).

## Discussion

While ART has transformed HIV-1 infection into a chronic treatable disease, virus persists in tissues that include the gut, lymph nodes, brain, spleen amongst other sites. The inability of ART to eliminate virus in these tissue sanctuaries remains the major obstacle towards a disease cure. Such a limitation is linked, in large measure, to continuous long-term infections in CD4 + memory T cells and less frequently in mononuclear phagocytes despite both directed host antiviral immunity and ART effectiveness. Thus, one may predict that, any or all steps towards HIV elimination must include precise targeted ART delivery, maintenance of vigorous immune control, effective blockade of viral growth and immune-based elimination of pools of infected cells or genome integrated proviral DNA. Even under these conditions, the presence of replication competent virus that allows low-levels of viral production and viral latency underscores employment of strategies that eliminate virus that is integrated but latent. Because of notable graft versus host disease in several humanized animal models, examinations for time periods measured in months are limited. In order to overcome the challenge of sustained human grafts in mice, we adopted NSG-humanized mice transplanted at birth with HSC. Both human myeloid and lymphoid lineages were successfully reconstituted in these mice and support the evaluations of HIV-1 persistence, treatment, and immune functions^17,30–38^. The sustained human grafts as confirmed by flow cytometry were viable and functional for more than 6 months, which provided a platform that allowed treatment interventions for prolonged time periods and a clear ability during ART to best establish a continuous latent HIV-1 reservoir in peripheral tissues and the brain and the noted immunological responses to the viral infection^12,17,29,36,37,39^. These previously published data support the successful use of humanized mice in studies of HIV/AIDS pathogenesis, therapeutics^40–42^, and treatment^12–14,16,18,29,43,44^. These studies, taken together, clearly provide a rationale for the scientific approaches taken in the current report^12,13,29,43,45–47^.

Therefore, our approaches towards evaluating viral cures have included the demonstrated ability of the drugs to reach sites of latent infection and to do so at significant levels^18,37,39,43,44^. Notably, the use of molecular tools can permanently eliminate the viral genome and preclude reactivation^20,21,24,48^. Thus, we suggest that the current successful outcome in achieving this goal in more than 30% of the infected experimental animals reflects the combinatorial use of a suitable animal model, control of viral set points, reach to the viral reservoirs, delivery and intracellular drug penetration of potent LASER ART, and the widespread employment of CRISPR-Cas9 gene editing. The latter enabled high efficiency excision of large fragments of the viral genome from anatomically privileged tissues. Results support the idea that maximal viral restriction must be first established prior to excision to achieve optimal viral editing by CRISPR-Cas9.

Current HIV-1 treatment patterns are defined by daily dosing of a combination of either two nucleoside reverse transcriptase inhibitors (NRTIs) and one integrase strand transfer inhibitor (INSTI), or two NRTIs and one nonnucleoside reverse transcriptase inhibitor. Rebound that follows affects both the number and function of CD4 + T cells leading to virus-associated co-morbid conditions. LASER ART was developed in an attempt to eliminate these limitations and was shown effective in establishing drug depots in macrophages with sustained antiretroviral activities and reductions in HIV-1 proviral load beyond ART alone^15,38,47,49–55^. The success in these prior studies led to the use of LASER ART in the current report in order to maximize ART ingress to cell and tissue sites of viral replication enabling the drugs to reach these sites at high concentrations for sustained time periods. The maintenance of slow drug release for times measured in weeks or longer provided optimal settings for viral excision^17,39,47^. ART particles coated with poloxamers enabled lipophilic hydrophobic prodrug crystals to readily cross cell and tissue barriers, aiding precision drug release to viral sanctuary sites^12–14,37,39,46^. These claims are reinforced by our prior studies demonstrating up to a 10-fold increase in viral restriction at two independent multiplicities of infection in CD4 + T cell lines with LASER ART when compared to conventional native drugs^12,13^. The advantages of LASER ART over native ART include rapid entry across cell membranes of both CD4 + T cells and macrophages (due to drug lipophilicity); accelerated antiretroviral drug entry into viral reservoir sites (including the brain, gut, lymph nodes, liver, bone marrow and spleen); increased intracellular drug delivery; and stable plasma concentrations observed over weeks to months. The ART were selected in order to produce sustained plasma concentrations 4X the protein-adjusted 90% inhibitory concentration. Notably, a single parenteral dose of NMDTG at 45 mg DTG equivalents/kg to mice provided plasma DTG concentration of 88 ng/ml at 56 days^32^. Liver, spleen and lymph node DTG concentrations were 8.0, 31.2 and 17.6 ng/g, respectively at 56 days following single treatment. At 14 days after NMABC and NM3TC given at 50 mg ABC or 3TC equivalents/kg to mice, ABC and 3TC plasma concentrations were 21 and < 7 ng/ml, respectively^12–14^. In summary, there was little to no residual ART in plasma or tissue at the time of animal sacrifice reflecting the robust viral rebound found in all infected mice treated with LASER ART alone. Further, significant efforts were made by us to demonstrate that one month after LASER ART was discontinued, viral rebound was detectable. All of this highlights the rationale for use of LASER ART over native ART. Most importantly, our research^12–14^ demonstrated that ART levels in plasma were undetectable during the period of measured viral rebound.

For elimination of proviral DNA, we chose the CRISPR-Cas9  gene editing platform and created a multiplex of gRNAs that caused cleavage of the viral genome at the highly conserved regions within the LTRs and the Gag gene. This strategy allowed for the removal of the large intervening DNA fragments across the viral genome and mitigated any chance for the emergence of virus escape mutants^20,23^. In support of this notion, results from cell culture and animal adoptive infection studies showed the absence of replication competent HIV-1 in the spleen and bone marrow of animals with no rebound that could be attributed to virus escape. Our choice for the use of AAV9 comes from earlier studies demonstrating the broad range tissue distribution of CRISPR-Cas9 in a mouse model^20^. Accordingly, the results in our current study verified the bioavailability of our gene editing molecule in various organs of the NSG humanized mice. No off-target effects were detected in in vivo deep sequencing and bioinformatics analysis that may be caused by the CRISPR-Cas9 editing strategy. Nevertheless, as expected naturally occurring cellular DNA variation was found in both untreated cells as well as in CRISPR-Cas9-treated cells. Examination of several potential target cellular genes performed on clonal cells expressing CRISPR-Cas9 by gene amplification and direct sequencing showed no mutations that may be caused by the presence of CRISPR-Cas9 in the cells.

Results from ddPCR showed 60% to 80% efficiency of viral DNA excision by CRISPR-Cas9. Of note, this approach quantified dual cleavage events that removed the DNA fragment spanning 5’LTR to 3’LTR, 5’LTR to gag, and gag to 3’LTR of the proviral genome. However, the occurrence of single site editing events that would permanently interrupt the viral DNA and potentially inactivate viral replication by introducing small InDel mutations at the cleavage sites are not included in this estimate^19^. Therefore, viral activation and rebound may not be observed under the conditions whereby excision efficiency is less than 100%. In recent studies, we demonstrated that inclusion of quadruplex of gRNAs for targeting Gag, Pol and two separate sites within the LTRs may yield slightly higher efficiency of viral DNA excision^24^. It is important to note that in those studies we employed a different mouse model, distinct AAV delivery system, and more importantly, different timelines for HIV-1 infection and viral DNA harvesting for conventional semi-quantitative PCR assays. In recent studies, we combined bioimaging, antiretroviral PK and sensitive tissue biodistribution studies to facilitate ART delivery into cell and tissue viral reservoirs in both humanized mice and non-human primates. These combined diagnostic and therapeutic modalities, coined theranostics, are being developed to facilitate effective HIV-1 elimination strategies in an infected human host^56^.

In conclusion, we employed a broad range of highly sensitive tests to evaluate HIV-1 elimination by LASER ART and AAV_9_-delivered CRISPR-Cas9 treatments. These included viral gene amplification, flow cytometry, adoptive viral transfers, on target and off target assays, and measures of viral rebound to demonstrate that combination therapies can safely lead to the elimination of HIV-1 infection. Results demonstrated that eradication of replication-competent HIV-1 present in infectious cell and tissue sites of infected animals can be achieved. Although reappearance of viremia in humans can be delayed^6^, rebound occurs on average 2 to 4 weeks after ART interruption^6,57,58^ and 5 to 11 days in humanized mice^59^. Despite the vigorous treatments offered, there was no evidence of outward untoward effects of any therapies (supplementary fig. 19)^12^ including the persistence of human adult lymphocytes in mouse plasma and tissue (Fig. 4). As such, these proof-of-concept results offer readily defined and realistic pathways toward strategies for HIV-1 elimination. Future studies are designed to improve delivery of agents to viral reservoirs and specifically eliminate residual latent viral infections. This is a first important step towards a longer journey for viral eradication.

## Methods

### Cell culture reagents

4-(2-Hydroxyethyl)-1-piperazineethanesulfonic acid (HEPES) buffer and ciprofloxacin were purchased from Sigma-Aldrich, St. Louis, MO. Diethyl ether, endotoxin-free water, gentamicin, acetonitrile (ACN), methanol, KH_2_PO_4_, bovine serum albumin (BSA), Triton X-100, LC-MS-grade water, and TRIzol reagent were purchased from Fisher Scientific, San Diego, CA. The TZM-bl reporter cell line (AIDS Reagent Program, Division of AIDS, NIAID, NIH, Bethesda, MD) and HEK-293T cells (the American Type Culture Collection (ATCC), Manassas, VA) were cultured in high glucose DMEM supplemented with 10% FBS and gentamicin (10 µg/ml). Jurkat (Clone E6–1, TIB-152™) cells were purchased from ATCC and cultured in Roswell Park Memorial Institute (RPMI) medium containing 10% FBS and gentamicin (10 µg/ml) (Sigma-Aldrich, St. Louis, MO). PBMCs were isolated from leukopaks by gradient centrifugation on Ficoll-Paque for 30 minutes at 600 g. PBMCs collected from the buffy coat were stimulated with PHA (5 µg/ml) for 24 h in RPMI with 10% FBS and gentamicin (10 µg/ml) supplemented with human recombinant interleukin-2 (rIL-2) at a concentration of 30 ng/ml ((STEMCELL Technologies, Seattle, WA). Fresh media was exchanged every 2–3 days.

### Cell culture HIV-1 infection

HEK-293T cells were transfected using CaPO_4_ precipitation method in the presence of chloroquine (50 µM) with 30 µg of pNL_4–3_-EGFP-P2A-Nef plasmid^22^ /2.5 × 10^6^ cells/100 mm dish. Next day, media were replaced; and 24 and 48 h later supernatants were collected, clarified at 1400 g for 10 minutes, filtered through 0.45 µm filter, and concentrated by ultracentrifugation for 2 h with 20% sucrose cushion. Viral pellets were resuspended in Hank’s Basic Salt Solution (HBSS) by gentle agitation overnight, aliquoted, and tittered in Jurkat cells by FACS for GFP expression. Jurkat cells were infected by spinoculation for 1.5 h, 32 °C in 500 µl inoculum containing 8 µg/ml polybrene then resuspended and left for 4 h then 500 µl of growth medium was added. Next day, cells were washed 3 times with phosphate-buffered saline (PBS) and re-suspended in growth medium.

### Design of gRNA, construction of CRISPR-Cas9 expression plasmid and the AAV_9_ vector

Bioinformatics design and cloning of LTR1 and GagD gRNAs into AAV-CMV-saCas9 vector was previously described^19,23^. Briefly, the Broad Institute gRNA designer tool (https://www.broadinstitute.org/rnai/public/analysis-tools/sgrna-design) was used to screen HIV-1_NL4–3 **or** ADA_ sequences for possible gRNA protospacer regions followed by saCas9 specific PAM: NGGRR(N). A pair of gRNAs showing the best predicted on-target (in HIV-1 genome) and the lowest off-target (in human genome) activities was selected: one targeting the HIV-1 LTR promoter region and the other targeting gag gene. The sequences of respective LTR1 and GagD gRNAs plus PAM were further cross referenced with Los Alamos HIV sequence database confirming high levels of conservation (>90%) across the HIV-1 sequences. Next, pair of oligonucleotides for each target site with 5′-CACC and 3′-AAAC Bsa1 overhangs was obtained from Integrated DNA Technologies (IDT, Coralville, Iowa, Table S1), annealed, phosphorylated, and ligated into *Bsa*I digested, dephosphorylated pX601-AAV-CMV:NLS-saCas9-NLS-3xHA-bGHpA;U6::BsaI-sgRNA (a gift from Feng Zhang via Addgene) (61591; Addgene). For multiplex gRNA cloning, the U6-LTR1-gRNAscaffold cassette from pX601-CMV-saCas9-LTR1 was amplified using T795/T796 primers (Table S1) and cloned using In-Fusion HD Cloning Kit (Clontech, Mountain View, CA) into *EcoR*I and KpnI linearized pX601-CMV-saCas9-GagD plasmid resulting in pX601-CMV-saCas9-LTR1-GagD AAV delivery vector. Finally, sequence verified plasmid was sent for packaging into AAV-9 serotype (Vigene Biosciences Inc., Milton Park Abingdon, UK). AAV_9_ was chosen as the vector for CRISPR-Cas9 delivery for its robust transduction efficiencies in multiple tissues including the central nervous system as significant putative reservoirs for HIV-1. The notion was to permit efficient AAV entry into all putative HIV-1 target tissues including the brain.

### HIV-1 infection of CD34 + humanized mice

NSG (NOD.Cg-Prkdc^scid^ Il2rgt^m1Wjl^/SzJ) mice were obtained from the Jackson Laboratories, Bar Harbor, ME and bred under specific pathogen-free conditions at the University of Nebraska Medical Center (UNMC) in accordance with the ethical guidelines set forth by the National Institutes of Health for care of laboratory animals. CD34 + HSC were enriched from human cord blood or fetal liver cells using immune-magnetic beads (CD34 + selection kit; Miltenyi Biotec Inc., Auburn, CA, USA). CD34 + cell purity was >90% by flow cytometry. Cells were transplanted into newborn mice irradiated at 1 Gy using a RS‐2000 × ‐Ray Irradiator (Rad Source Technologies, Buford, GA). Cells were transplanted by intrahepatic (i.h.) injection of 50,000 cells/mouse in 20 μl phosphate-buffered saline (PBS) with a 30-gauge needle. The experiments shown in Fig. 2–6 were from human fetal liver cells were isolated from a single donor. In the study described in Figs. 7 and 8, cord blood-derived HSC were obtained from two donors. Mice from a single donor were used for all dual treatment mice. Humanization of the animals was affirmed by flow cytometry^31,60^ for the presence of human CD45 and CD3 positive blood immune cells, as shown in Fig. 4. At 18 weeks of age, 25 NSG-hu mice were infected intraperitoneally (i.p.) with HIV-1_NL4–3_^32,36^ at 10^4^ tissue culture infective dose_50_ (TCID_50_)/ml and sacrificed at days 1, 3, 7, and 14; *n* = 5 at each time point. Five control-uninfected animals were included in all test evaluations. Levels of viral RNA copies/ml were analyzed with the automated COBAS Ampliprep System V2.0/Taqman-48 system (Roche Molecular Diagnostics, Basel, Switzerland)^30,31^. For this assay, 100 μl of mouse serum was diluted to 1 ml with sterile filtered normal human serum. The detection limit of the assay after dilution is 200 viral RNA copies/ml. Although the eclipse phase for viral infection in humans remains variable^61^, the viral loads and CD4 + T cell depletion levels observed in our infected humanized mice are in point of fact reflective of the disease course in an infected human host. Indeed, only after weeks of infection we do observe significant cell loss^12,17,29,37,50^. These findings can be viewed as an affirmation of the model including CD4 + T cell timed-restorations seen after ART as is seen in humans.

### Nanoformulated antiretroviral drugs

DTG, 3TC and ABC were generous gifts from ViiV Healthcare, Research Triangle Park, NC. RPV was purchased from Hangzhou Bingo Chemical Co., Ltd, Hangzhou, China. Antiretroviral prodrugs and their polymer encasements were performed as previously described^12–14^. Myristoylated modifications for DTG, 3TC, and ABC were made (referred to as MDTG, M3TC, and MABC) to enhance the incorporation into poloxamer 407 (P407) nanoparticles, while RPV was encased solely by poloxamer 338 (P338) in unmodified form using high pressure homogenization to form crystalline nanoformulated drugs. Particle size, polydispersity index, and zeta potential were determined by dynamic light scattering using a Malvern Nano-ZS (Malvern, Worcestershire, UK)^49^. Final drug concentrations in the nanoformulation suspensions and injection solutions were determined by HPLC-UV/Vis and UPLC-MS/MS. A 40–50 μl volume for each nanoformulation combination (NMDTG/NRPV and NM3TC/NMABC) was administered by intramuscular (IM) injection in opposing thigh muscles of the mice.

### Antibodies

For flow cytometric analysis, we used a panel of antibodies (all from BD Biosciences, San Jose, CA) comprised of FITC-conjugated mouse anti-human CD45 (catalog #555482), Alexa Fluor 700-conjugated mouse anti-human CD3 (catalog #557943), APC-conjugated mouse anti-human CD4 (catalog #555349), and BV421-conjugated mouse anti-human CD8 (catalog #562428), PE-conjugated mouse anti-human CD14 (catalog #555398), and PE-Cy5-conjugated mouse anti-human CD19 (catalog #555414) antibodies. For immunohistochemical staining, we used monoclonal mouse anti-human HIV-1p24 (clone Kal-1, M0857, Dako, 1:10), monoclonal mouse anti-human leukocyte antigen (HLA-DR; clone CR3/43, Dako, 1:100), and the polymer-based HRP-conjugated anti-mouse EnVision + secondary antibodies were purchased from Dako (Carpinteria, CA). Peripheral blood was collected from the submandibular vein into ethylenediaminetetraacetic acid (EDTA)-coated tubes or by cardiac puncture at the study end. Blood leukocytes were tested for human pan-CD45, CD3, CD4, CD8, CD14, and CD19 markers as six-color combinations using LSR-II FACS analyzer (BD Biosciences). Antibodies and isotype controls were obtained from BD Pharmingen, San Diego, CA, and staining was analyzed with a FlowJo (BD Immunocytometry Systems, Mountain View, CA). The gating strategy is shown in supplementary fig. 20. Results were expressed as percentages of total number of gated lymphocytes. The percentages of CD4 and CD8 positive cells were obtained from human CD3 + gate^17^. We used absolute counts of human CD45 + cells to normalize each of the human cell data sets. Equivalent numbers of total blood cells/mouse were used at each time point.

### Immunohistochemistry (IHC) examinations

Spleen, lung, liver, and lymph nodes were perfused with PBS followed by 4% paraformaldehyde and then post-fixed overnight and processed for paraffin embedding. Five-micron thick sections were cut from the paraffin blocks, mounted on glass slides, and labeled with mouse monoclonal antibodies (Dako) for HLA-DQ/DP/DR (clone CR3/43, 1:100) and HIV-1p24 (1:10). The polymer-based HRP-conjugated anti-mouse Dako EnVision system was used as a secondary detection reagent and developed with 3,3′-diaminobenzidine (DAB). All paraffin-embedded sections were counterstained with Mayer’s hematoxylin. Deletion of primary antibodies or using mouse IgG served as controls. Images were obtained with a Nikon DS-Fi1 camera fixed to a Nikon Eclipse E800 (Nikon Instruments, Melville, NY) using NIS-Elements F 3.0 software.

### Nucleic acid extractions and qPCR assays

In studies presented in Fig. 2–8, total viral nucleic acids (RNA and DNA) were extracted from the spleen, bone marrow cells, lung, gut, liver, kidney, and brain using a Qiagen Kit (Qiagen, Hilden, Germany) according to the manufacturer’s instructions. Total cellular DNA obtained from the HIV-1 infected cell line ACH2 served as a positive control and standards, while human genomic DNA obtained from uninfected NSG-hu mice served as a negative control. Cell-associated HIV-1 RNA and DNA were quantified by real-time qPCR and droplet digital PCR (ddPCR) assays. Because of extremely low numbers of latently-infected human cells in HIV-infected NSG-hu mice after long-term ART, detection of total HIV-1 DNA,  required two rounds of PCR amplification. The first round of PCR was performed on a conventional PCR machine (T100 Thermal Cycler, Biorad, CA) in 25 μl of PCR reaction mix containing 500 ng of template and 50 ng each of both primers annealing to HIV-1 gag region and the reaction conditions are as follows: 94 °C for 3 min, followed by 15 cycles of 94 °C for 30 s, 55 °C for 30 s, and 72 °C for 1 min. The product of the first PCR was subsequently used as a template in the second semi-nested real-time PCR amplification performed on the ABI Step One Plus real-time PCR machine (Applied Biosystems, Foster City, CA) using TaqMan detection probe and primers^30^. Two μl of the first PCR product was diluted to 50 μl with PCR master mix containing two primers at 0.2 μM each and 0.2 µM TaqMan dual-labeled fluorescent probe. Real-time PCR settings were as follows: 50 °C for 2 min, then 95 °C for 10 min, followed by 40 cycles of 95 °C for 15 s, and 60 °C for 1 min. The amplicon sizes are 221 bp for the first round of PCR and 83 bp for the second round (real-time) PCR. DNA extracted from ACH2 cells containing one integrated copy of HIV-1 per cell was used as standard in serial 10-fold dilutions with HIV copy numbers ranging from 10^1^ to 10^5^ DNA copies/reaction^36,37^. Integrated DNA (iDNA) provirus was quantified using an adapted Alu-gag PCR assay as described by Agosto et al.^62^ with modifications for the second round of PCR, following prior published methods^63^. Briefly, samples underwent a first-round PCR amplification (95 °C for 2 min; 20 cycles of 95 °C for 15 s, 50 °C for 15 s, and 72 °C for 150 s) using 100 nM Alu and 600 nM *gag* reverse primers. Five μl of the first-round product were amplified in a nested protocol using the assay for HIV-1 gag gene (second PCR primers and probe), as described above. First-round PCR included 3 replicates using only gag reverse primer (gag only) to serve as background un-integrated control. Integration levels per cell were calculated by subtracting gag-only signals from the Alu-gag quantification. Semi-nested real-time RT-PCR on HIV-1 RNA was performed as described^36,37^. The eluted cellular RNA was first subjected to DNase treatment to remove HIV-1 DNA to avoid the interference with the quantitation. For reverse transcription assay, random hexamers were used as primers and SuperScript III (Invitrogen, MA) to synthesize first-strand cDNA at 42 °C for 60 min. cDNA was used for the unspliced (usRNA) assay. Two rounds of PCR were performed under the same PCR conditions as described for total viral DNA. For the usRNA assay, real-time PCR was run for 45 cycles and same primers and fluorescent probe as for the total viral DNA assay were used. Human CD45 species-specific primers and probes were obtained from Thermo-Fisher Scientific (USA) (cat. no. 433182 for Hs0036534_g1).

For viral excision testing, frozen tissues sent to Temple University from the University of Nebraska Medical Center were homogenized using Bullet Blender homogenizer (Next Advance, Averill Park, NY) using bead combinations and settings specific for every tissue according to manufacturer’s protocols. T1 buffer from NucleoSpin Tissue kit (Macherey-Nagel, Duren, Germany) was used for homogenization/initial lysis followed by over-night proteinase K digestion. Extraction of genomic DNA was completed according to the manufacturer’s protocol. For standard PCRs (supplementary Table 1), 500 ng of extracted DNA were subjected to PCR using Fail Safe PCR kit and buffer D (Epicentre, Madison, WI) under the following PCR conditions: 94 °C 5 min, 30 cycles (94 °C 30 s, 55 °C 30 s, 72 °C 30 s), 72 °C 7 min using 1st round primers followed by nested PCR using diluted 1st round PCR reaction. Nested PCR products were subjected to Sanger sequencing directly if only one amplicon population was detected by agarose gel electrophoresis. For multiple amplicons detected, in order to investigate the composition of HIV excision, each amplicon population was separated and purified from an agarose gel electrophoresis and then cloned into TA vector (Invitrogen, Carlsbad, CA). Plasmid DNA containing excised HIV amplicon was purified from each bacterial colony for Sanger sequencing (Genewiz, South Plainfield, NJ). HIV-1 DNA was quantified using TaqMan qPCR specific for HIV-1 pol and env genes and cellular beta-globin gene as a reference (supplementary Table 1). Prior to qPCR, genomic DNA was diluted to 10 ng/µl and then 5 µl (50 ng) were taken per reaction/well. Reaction mixtures were prepared using Platinum Taq DNA Polymerase (Invitrogen) according to a simplified procedure^64^. Standard was prepared from serial dilutions of U1 cells genomic DNA since it contains two single copies of HIV-1 provirus per diploid genome equal to beta-globin gene copy number. qPCR conditions: 98 °C 5 min, 45 cycles (98 °C 5 min, 45 cycles (98 °C 15 s, 60 °C 30 s with acquisition, 72 °C 1 min). Reactions were carried out and data analyzed in a LightCycler96 (Roche, Basel, Switzerland). For RT-PCR, TRIzol reagent (Ambion, Austin, TX) was used for initial RNA extraction followed by clean up using RNeasy kit (Qiagen, Hilden, Germany) with DNAse I digestion in the extraction column. Total 0.5ug of RNA was used for M-MLV reverse transcription (Invitrogen). For gRNA expression screening specific reverse primer (pX601gRNA scaffold/R, supplementary table 1) was used in RT reaction followed by standard PCR using target LTR 1 or GagD sense oligos as forward primers (supplementary table 1) and agarose gel electrophoresis. For checking saCas9 mRNA expression oligo-dT primer mix was used in RT and cDNA was subjected to PCR using saCas9 specific primer pairs and β-actin as a reference (supplementary table 1). Sanger sequencing results were analyzed using Clustal Omega (EMBL-EBI) multiple sequence alignment tool and Sequence Scanner Software 2 (Applied Biosystems).

### ddPCR for detection of HIV-1 nucleic acids

ddPCR was performed based on the water–oil emulsion droplet technology, using the ddPCR™ Supermix for Probes reagents in the QX200™ Droplet Digital™ PCR system (Bio-Rad Laboratories, Hercules, CA). For quantification of HIV-1 DNA, the eluted cellular DNA was PCR-amplified^36,37,63^ using Taqman sets targeting the HIV-1 LTRs, gag and pol genes and as a reference mouse or human beta-actin (supplementary Table 1). A total of 100–200 ng DNA from each tissue was used as template for ddPCR amplifications with the same thermal cycling conditions used for real-time q-PCR detection. Data acquisition and analysis were done using QX200 droplet reader and QuantaSoft™ software provided with the instrument. The genomic DNA extracted from a total of 50,000 cells including human and mouse cells was used as template for each ddPCR analysis. The reduction of Gag represents a deletion between 5’LTR and Gag or 5’LTR to 3’LTR, while a reduction in Pol represents the excision between Gag to 3’LTR or 5’LTR to 3’LTR. However, a single LTR will always remain detectable in all three conditions. Thus, we used the ratio of Gag or Pol to LTR to estimate the excision efficiency. For example, in mouse M4349 (Fig. 6e), the ratios of Gag/LTR and Pol/LTR are 19.7% (15 cells with detectable gag out of 76 cells with detectable LTR) and 19.5%, respectively, in the genomic DNA extracted from the spleen of the treated mice. Thus, the excision efficiencies of 5’LTR to Gag and Gag to 3’LTR were estimated to be about 80% for both (100%-19.7% or100% - 19.5%). In the spleen of the same mouse, the AAV9 transduction efficiency was calculated as high as 1.03 AAV vector copies/cell in the total population including both human graft and mouse host cells (Fig. 6g). In another mouse M4346 (Source Data file for Fig. 6e), we demonstrated that the excision occurred mainly in Gag to 3’LTR because the ratio of Pol/LTR is 38.4% while Gag/LTR is 89.5%. Thus, the excision efficiency was estimated at 61.6% in 5’LTR to Gag and 10.5% in Gag to 3’LTR. Nonetheless, the presence of 2 LTRs in an uncut HIV proviral DNA was not considered in order to simplify the estimate.

### RNAscope

Viral RNA was detected as single brown dots or cluster of dots in 5 μm thick paraffin embedded spleen and lymph node tissue sections using antisense probe V-HIV1- Clade-B (Cat no 416111) targeting 854–8291 bp of HIV-1_NL4–3_^65^. Human peptidylprolyl Isomerase B (PPIB) was used as positive control for the spleen tissue analyzed (images were captured at 40-x magnification). All reagents are from Advanced Cellular Diagnostics, Newark, CA.

### Viral recovery

PBMCs obtained from leukopaks from HIV-1,2 seronegative donors were stimulated with PHA and IL-2 and co-cultured with human bone marrow or spleen cells recovered from 3 groups of CD34 + HSC- NSG mice that included HIV- 1 infected, infected and LASER ART treated, and LASER ART and AAV_9_-CRISPR-Cas9-treated mice. PBMCs were used in assays after a 3-day treatment maintained in 10% RPMI with 30 U/ml of IL-2 then co-cultured with human bone marrow or spleen cells at concentrations of (1:5)^66–68^. Cells were harvested eight days later for HIV-1 DNA (A) and RNA (B) using semi-nested real-time qPCR assay and supernatant fluids assayed for reverse transcriptase activity for up to day-14. Data are expressed as total HIV-1 DNA (A) or RNA (B) copies/10^6^ human CD45 + cells. One of the two dual-treated animals was tested and confirmed viral sterilization. Viral rescue was observed in other groups of animals tested.

### Adoptive transfers

Splenocytes and bone marrow cells (8–10 × 10^6^) were harvested at the time of sacrifice from NSG-hu mice that were HIV-1_ADA_ infected with and without LASER ART and AAV_9_-CRISPR-Cas9. The cells were adoptively transferred into unmanipulated 18-week old CD34 + HSC-NSG mice. Cell counts and viability tests were determined by both trypan blue and live/dead stains on the TC-20 automated cell counter (Bio-Rad). Cells were injected IP into mice and monitored for an additional 4 weeks. These experiments were performed to cross validate eradication of viral infection that could occur from latent reservoirs and not detected by either qPCR, RNAscope, and ddPCR assays. Viral load was measured from blood samples of the adoptively transferred mice using automated COBAS Ampliprep System V2.0/Taqman-48 system, and immune cell profiles (CD4 and CD8 + T cells by flow cytometry) recorded, in parallel. Residual virus from all humanized mice tissues was examined by qPCR and ddPCR assays. Virus was not detected in plasma or tissues from two adoptively transferred animals (mice M3319 and M3336).

### Off-target analyses

TZM-bl cells were plated in 6 well plates at 1 × 10^5^ cells/well and co-transfected using Lipofectamine 2000 reagent (Invitrogen) with 1 µg of control pX601-AAV-CMV:NLS-SaCas9-NLS-3xHA-bGHpA;U6::Bsa1-sgRNA (Addgene catalog #61591) or 1 µg of pX601-LTR1-GagD (20) plasmid together with 0.2 µg of pKLV-U6gRNA(Bbs1)-PGKpuro2ABFP (Addgene catalog #50946) to provide puromycin selection marker. The next day, cells were transferred into 100-mm dishes and cultured in the presence of puromycin (Sigma) at concentration 1 µg/ml. After two weeks, surviving clones were isolated using cloning cylinders (Corning, Corning, NY, USA). Genomic DNA was prepared from each single cell clone and LTR specific PCRs followed by gel purification; TA cloning and Sanger sequencing were performed. The clones showing the presence of on target CRISPR-Cas9 induced InDel mutations at target LTR 1 site in integrated HIV-1 LTR sequence (*n* = 6) together with two control clones were selected for further in vitro off target analysis. The list of potential OFF target sites in human genome for HIV-1 target LTR 1 and GagD was created using Benchling CRISPR design tool (https://benchling.com/, supplementary  table 2). A total of three potential OFF target sites were chosen (the top scorer plus two top gene specific potential off target sites, see supplementary  table 2, highlighted in yellow) for PCR based screening in selected single cell clones. The potential OFF target regions were PCR-amplified, cloned into TA vector, and performed Sanger sequencing (3–6 sequences/single cell clone/single OFF target) (supplementary table 3).

The genetic variation analyses among the three treatments were performed through the next generation sequencing (by the Novogene NGS facility) and bioinformatics tools for four sample animals, one animal from the LASER ART, one animal from CRISPR-Cas9- and two no-rebound animals from the LASER ART/CRISPR-Cas9 groups. The main objective was to detect the possible CRISPR-Cas9 off-target sites. Besides this, some genetic variations such as single nucleotide polymorphisms (SNP), insertion-deletions (InDels), structural variants (SVs), and copy number variants (CNVs) were analyzed for those four animals; and the results are in supplementary tables 4–5. After a thorough quality control step, the resulting paired-end short-reads were mapped to the human reference genome (Human_G1K_V37) utilizing Burrows-Wheeler Aligner (BWA) algorithm. For the animals M4356 (CRISPR-Cas9), M4348 and M4349 (LASER ART + CRISPR-Cas9), and M3539 (LASER ART), the 8 coverages were reported to be 92.01%, 91.97%, 92.01%, and 91.92%, while the sequencing depths were 36.08, 63.11, 45.22, and 15.41, respectively.

### Excision efficiencies and hierarchal clustering

The excision efficiencies for each animal, tissue, and HIV-1 gene segment were calculated as the ratio of the number of the sequencing-verified PCR product to all members in each group with denoted experimental conditions (i.e. treatments, cells and tissues shown in Figs. 3, 4, 5, 6 and supplementary fig. 4). Defined such that the excision efficiencies can be viewed as frequentist probabilities, i.e. the ratio of the frequency of occurrence of the event of interest to the total number of experimental repeats. This interpretation of excision efficiencies provides the user with a predictive value, as they can be used to set a prior expectation on the success rate of each treatment (LASER ART, CRISPR-Cas9, and LASER ART plus CRISPR-Cas9) in excising the desired segments of HIV-1 gene in the studied tissues and further to relate that to the likelihood of cure. Hierarchical clustering was performed on the efficiency values of truncation events under different treatments and across different animals, tissues, and HIV-1 gene segments. Once the excision efficiencies were calculated under different combinations of experimental conditions, the hierarchical clustering scheme was employed to group the efficiency values into a multilevel cluster tree represented by a dendrogram. This hierarchical clustering heat map may offer a predictive capability for viral elimination. Bioinformatics analysis of human genome sequence data identified human genome sites that could serve as off targets for gRNAs designed for editing HIV-1 DNA. The corresponding efficiency values were listed in heat-map table to make the clusters visually detectable. To this end, three combinations were considered: i) excision probabilities of different HIV-1 segments in 6 different tissues of animals undergoing antiretroviral treatment, CRISPR-Cas9 mediated editing, and the combined treatments; ii) excision probabilities of different segments in different animals under the three treatments; and iii) probabilities of observing at least one positive band for each specified tissue in all animals. Clusters of supplementary figs. 10–16 also include additional conditions of cure and qPCR data to identify which animals experienced complete cure and highest viral genome eradication.

### Study Approval

All experimental protocols involving the use of laboratory animals were approved by the University of Nebraska Medical Center (UNMC) Institutional Animal Care and Use Committee (IACUC) ensuring the ethical care and use of laboratory animals in experimental research. Human blood cells were isolated by leukapheresis from HIV-1/2 and hepatitis seronegative donors and were deemed exempt from approval by the Institutional Review Board (IRB) of UNMC. Human CD34 + hematopoietic stem cells were isolated from human fetal liver and umbilical cord blood and are exempt from UNMC IRB approval.

### Statistics

The data were analyzed using GraphPad Prism 7.0 software (La Jolla, CA) and presented as the mean ± the standard error of the mean (SEM). Experiments were performed using a minimum of three biologically distinct replicates. Sample sizes were not based on power analyses as the efficiency of HIV-1 elimination was not known nor could it be projected. For comparisons of two groups, Student’s *t* test (two-tailed) was used. Tissue drug levels, HIV-1 RT activity, HIV-1p24 antigen staining, T-cell populations, viral RNA and DNA, and viral load were analyzed by one-way ANOVA with Bonferroni correction for multiple-comparisons. For studies with multiple time points, two-way factorial ANOVA and Bonferroni’s post-hoc tests for multiple comparisons were performed. Extreme outliers beyond the 99% confidence interval of the mean and 3-fold greater than the SEM were excluded. Significant differences were determined at *P* *<* 0.05.

### Reporting Summary

Further information on research design is available in the Nature Research Reporting Summary linked to this article.

## Supplementary information

Supplementary Information

Reporting Summary

Source Data

## Data Availability

The sequencing data reported in this paper are included in the Source Data file. The source data underlying Figs. 4d, 5b, c 6b, c, e, f 7f, and 8c, and Supplementary Figs. 1, 4–12 and 17 are provided as a Source Data file. All other relevant data supporting the key findings of this study are available within the article and its supplementary files or from the corresponding authors upon reasonable request.
