# Supplementary material for: Sequential LASER ART and CRISPR Treatments Eliminate HIV-1 in a Subset of Infected Humanized Mice
Source: Nat Commun. 2019 Jul 2;10:2753. doi: 10.1038/s41467-019-10366-y (PMC6606613; doi:10.1038/s41467-019-10366-y)
Supplement: Supplementary file 1 — Supplementary Information [file 41467_2019_10366_MOESM1_ESM.pdf]

Supplementary materials

Sequential LASER ART and CRISPR Treatments

Eliminate HIV-1 in a Subset of Infected Humanized Mice

Dash et al.

Supplementary Figures with Legends

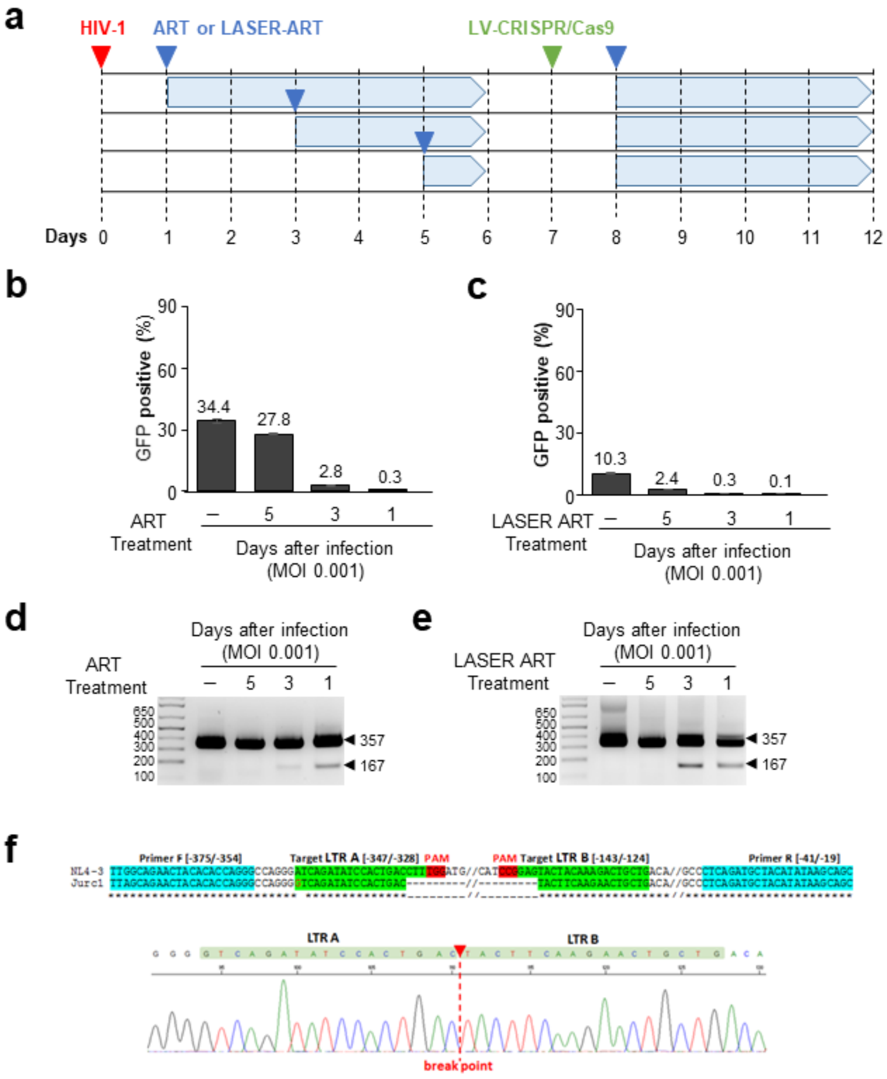

Supplementary Figure 1.

Combined effect of ART and CRISPR-Cas9 on HIV-1 infection of Jurkat T cell line. (a) Jurkat cells were infected with HIV-1NL4-3-GFP-P2A-Nef at multiplicity of infection (MOI) of 0.001. Next, cells were divided into four groups: one DMSO control and three treated with the cocktail of four antiretroviral drugs (ARVs) at the concentrations of 5x EC90 values (DTG at 11.1 ng/ml, RPV at 3.3 ng/ml, 3TC at 17.2 µg/ml and ABC at 8.3 µg/ml). A replicate experiment was performed using LASER ART formulations at the doses 5x EC90 values (NMDTG at 16.7 ng/ml, NRPV at 3.3 ng/ml, NM3TC at 32.9 µg/ml and NMABC at 14.4 µg/ml). ART or LASER ART treatment was started at days 1, 3 or 5 after infection and fresh drugs were added daily. At day 6 of infection, the drugs were removed to allow efficient transduction of cells with lentivirus expressing CRISPR-Cas9, which was applied at day 7. At day 8 ARVs were added back and continued for another 4

days. At day 12 post-infection, cells were collected, genomic DNA was extracted and analyzed by PCR for CRISPR-Cas9 mediated cleavage of viral LTR sequences. (b) Quantification of the level of infection at day 7. Cells were fixed with 2% PFA and FACS analysis was performed to measure GFP expressing population for HIV infection/replication in vitro. (c). Similar to panel b with exception that cells were treated with LASER ART. Results from standard PCRs of genomic DNA obtained from infected and treated T cells. The presence of full length LTR (357bp) and truncated, CRISPR-Cas9 induced products (167bp) was examined in cells that received ART (d) or LASER ART (e) and aligned to HIV genome after Sanger sequencing (f). Results of the truncated PCR product obtained after purification from the agarose gel and TA cloning. gRNAs target sequences are shown in green, PAM sequences in red and PCR primers in blue. Below is a representative example of Sanger sequence tracing of truncated product. The HIV-1 LTR sequence was cleaved by Cas9 at target sites LTR A and LTR B and then re-joined, resulting in deletion of 190bp proviral DNA segment. The double cleaved/end-joined site is shown as a breaking point in red. Sanger sequencing results are included in FASTA format in the Source Data file.

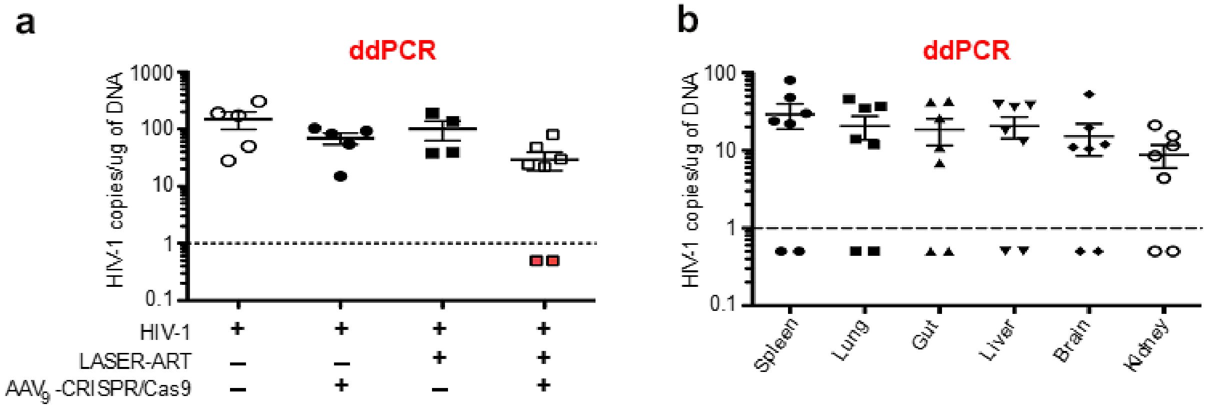

Supplementary Figure 2.

Analysis of HIV-1 copies in humanized mice tissues using ultra-sensitive ddPCR. Ultrasensitive droplet digital PCR (ddPCR) with sensitivity of detecting 1-2 copies was used to detect viral DNA in spleen of the infected animals belonging to 4 groups, control infected, LASER ART or CRISPR-Cas9 alone treated and dual treatment (LASER ART and CRISPR-Cas9) (a) and the various organs of the two mice with no viral rebound (b). Note that two animals in the double treatment group (group 4, M4346 and M4349) showed complete viral elimination in spleen and the other tissues tested (lung, liver, gut, brain and kidney).

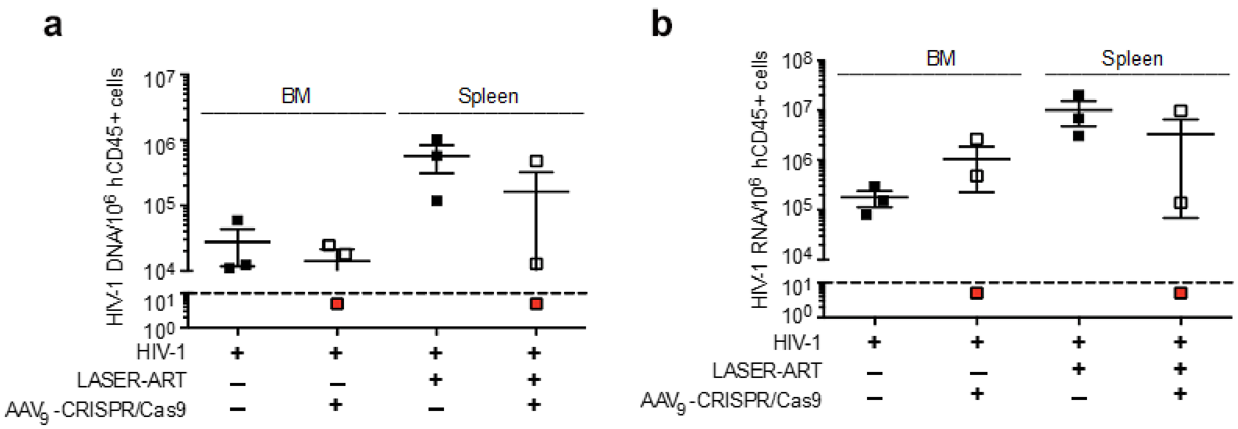

Supplementary Figure 3.

Viral recovery assay using cell co-cultivation. Splenocytes and bone marrow (BM) cells were isolated from HIV-1 infected mice with or without prior LASER ART and CRISPR-Cas9 treatments then co-cultivated with PHA/IL-2 stimulated human peripheral blood mononuclear cells. Cells were harvested 12 days after co-cultivation for HIV-1 DNA (a) and RNA (b) and rebound virus examined using highly sensitive semi-nested real-time q-PCR assay. Data are expressed as total viral copies/10^6 human CD45+ cells. Dual LASER ART and CRISPR-Cas9 treatment resulted in no detection of viral nucleic acids, which was also confirmed by reverse transcriptase assay of culture supernatants. Virus was detected in all other groups of animals.

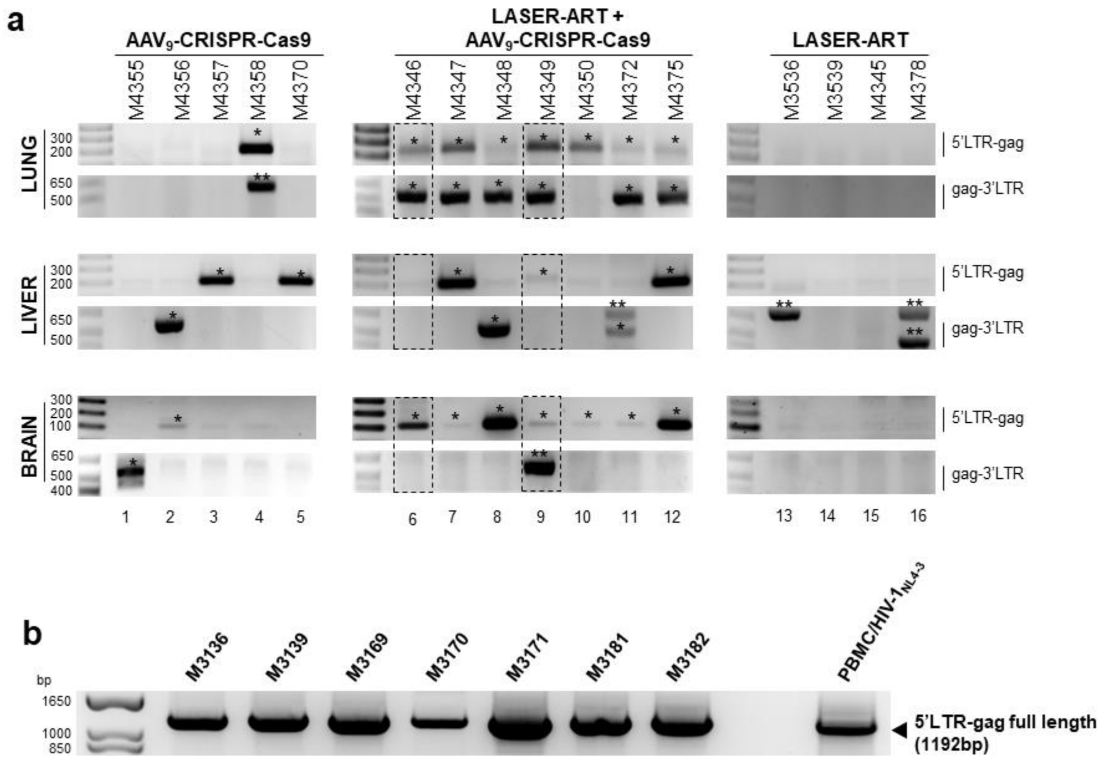

Supplementary Figure 4.

Excision of the viral DNA fragments by CRISPR-Cas9 in tissues from HIV-1 infected humanized mice with and without treatments with LASER ART. Results from standard PCRs of genomic DNA obtained from lungs, livers and brains of treated animals. The presence of truncated CRISPR-Cas9 induced products (193bp for 5'LTR-Gag and 523bp for Gag-3'LTR) were tested. *CRISPR-Cas9 mediated excision products. **Non-related. All bands were verified by Sanger sequencing and sequences are included in FASTA format in the Source Data file (a). Detection of HIV-1 DNA sequence corresponding to the 5'-LTR to Gag gene in lung from LASER ART treated animal where no excision is detected (b).

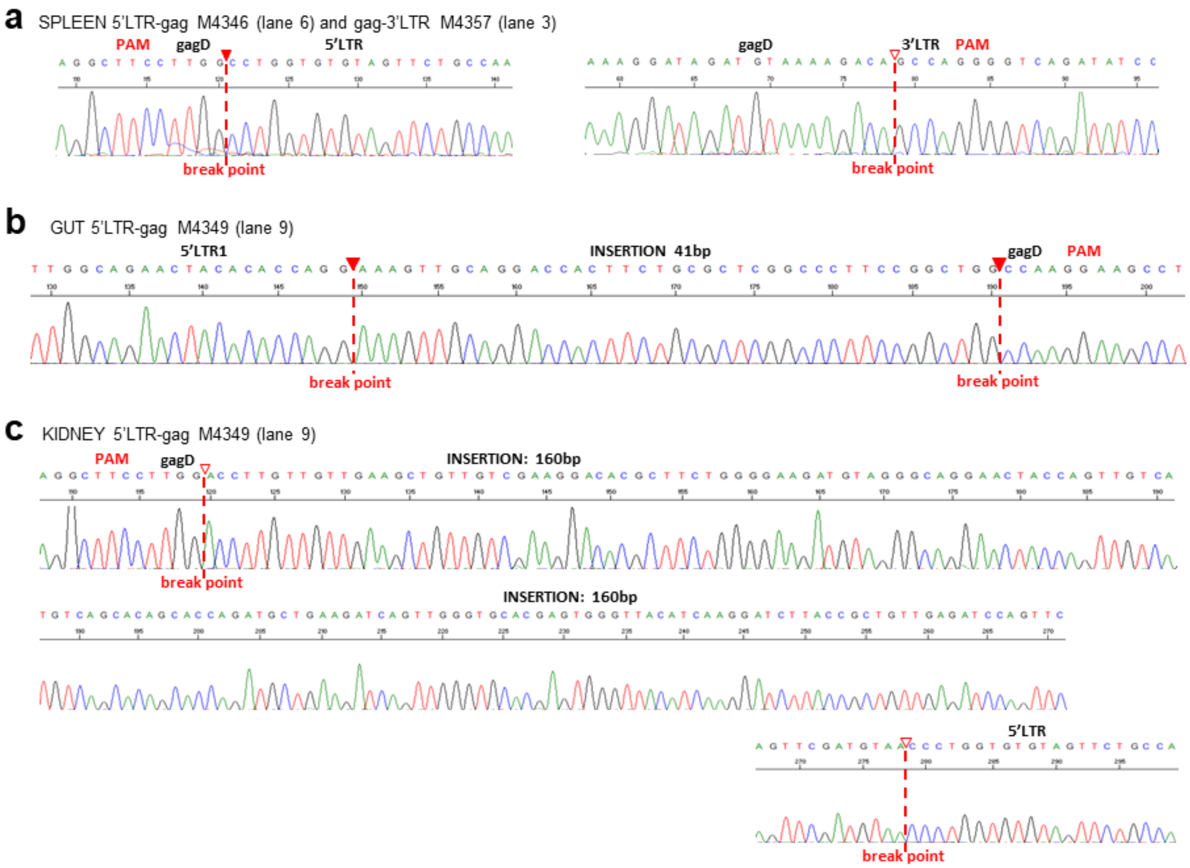

Supplementary Figure 5.

Sanger sequencing shows truncated, CRISPR-Cas9 excised HIV-1 genomes in treated and infected humanized mice. (a) Representative examples of canonical, InDel free, CRISPR-Cas9 induced, double cleaved/end-joined HIV-1 genome truncations observed in majority of the tissues of CRISPR-Cas9/gRNA treated animals. On the left, result obtained from the spleen of mouse M4346 using 5'LTR-Gag specific primers and on the right sequence from the spleen of mouse M4357 using Gag-3'LTR specific amplification. (b) Verification of the presence of 41bp insertion at the CRISPR-Cas9 mediated cleavage site in the viral sequence observed in gut sample from mouse M4349. (c) Sequence of the longer, 160bp insertion found at the Cas9 cleavage site in the kidney sample from the same mouse M4349. Sanger sequencing results are included in FASTA format in the Source Data file for Fig. 6bc.

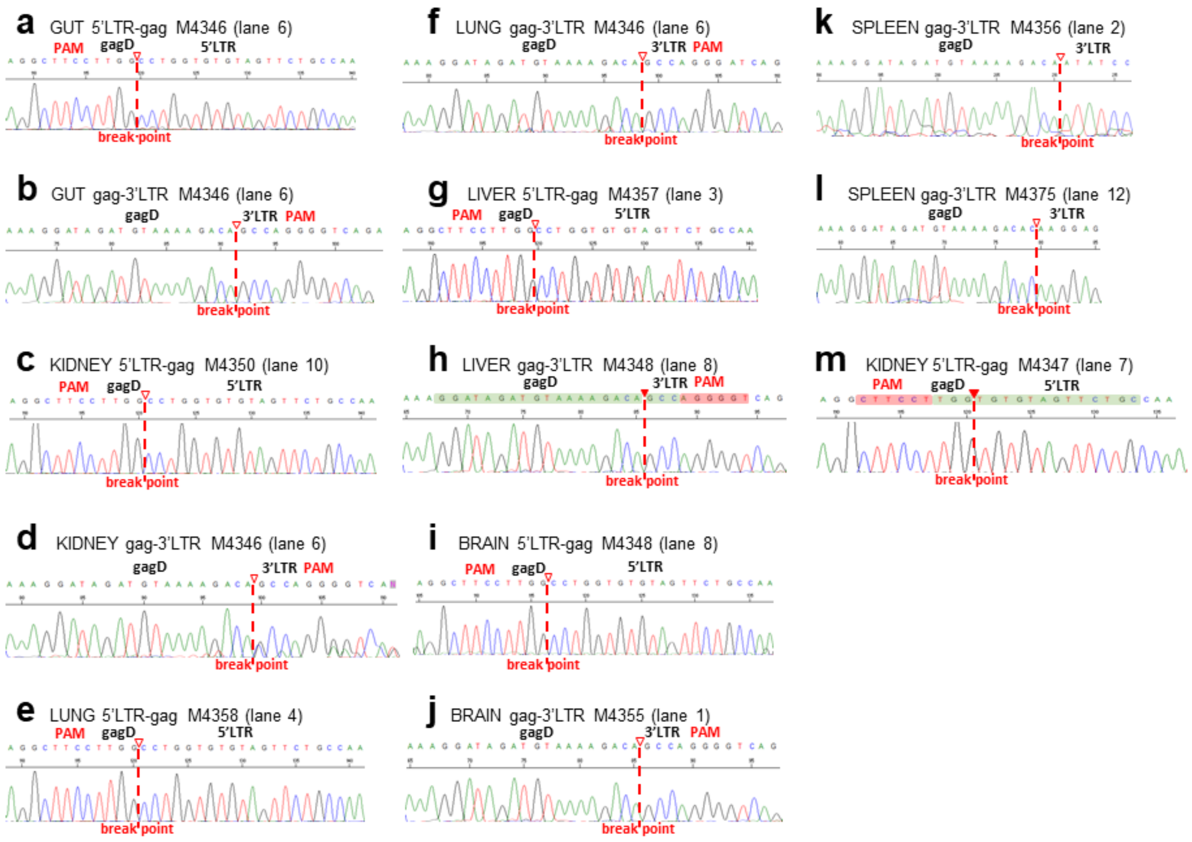

Supplementary Figure 6.

Sanger sequencing results of the truncated, CRISPR-Cas9 excised HIV-1 genomes in diverse tissues of infected and treated humanized mice. Representative examples of canonical, InDel free, CRISPR-Cas9 induced, double cleaved/end-joined HIV-1 genome truncations observed in majority of the tissues of Cas9/gRNA treated humanized mice (a, b for gut, c, d for kidney, e, f for lung, g, h for liver and i, j for brain). InDel mutation detected at the cleavage/end-joining sites in several tissues are shown in k, l for spleen, m for kidney. Sanger sequencing results are included in FASTA format in the Source Data file for Fig. 6bc.

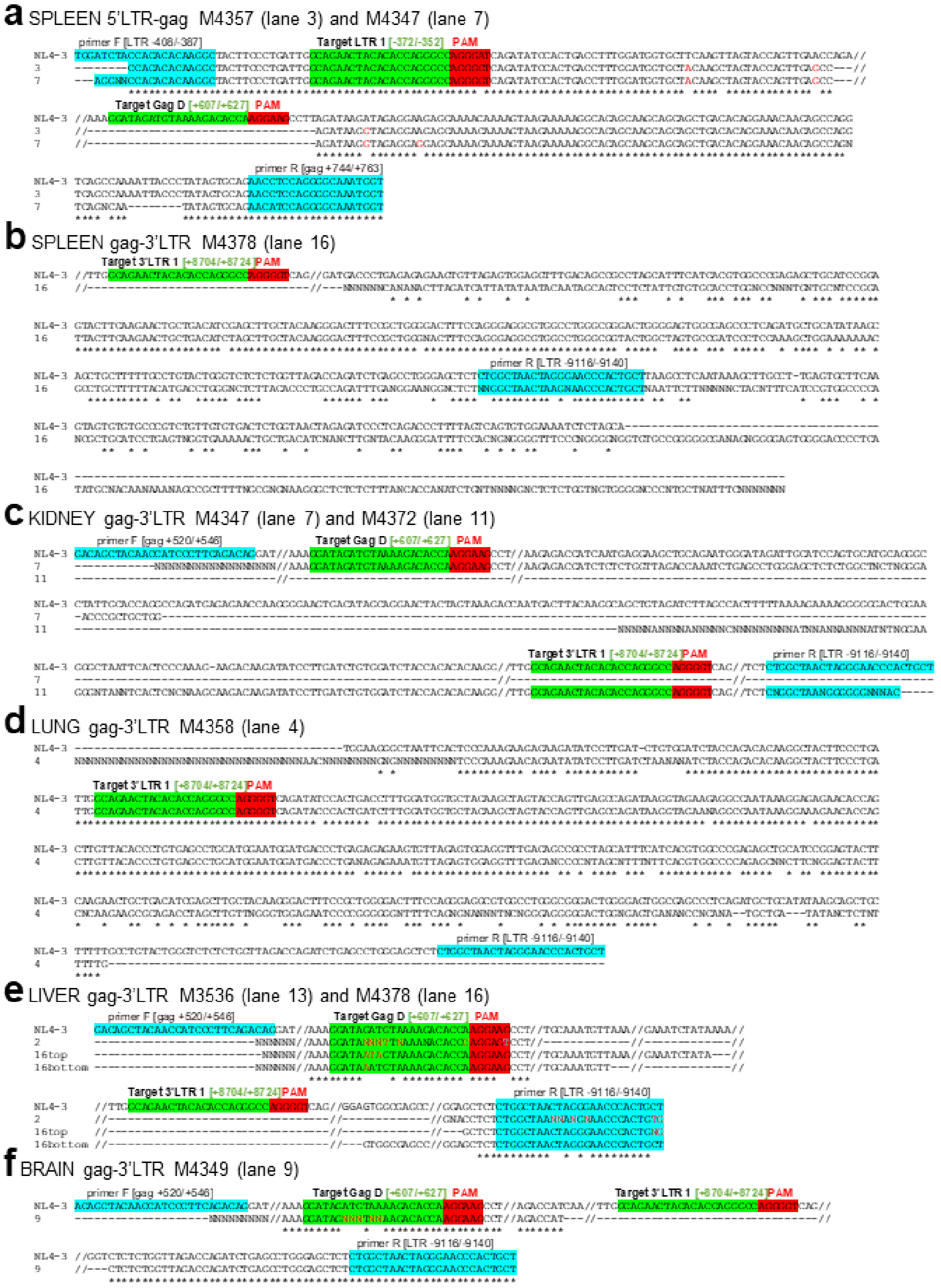

Supplementary Figure 7.

Sanger sequencing results of truncated HIV- 1 amplicons detected in few tissue samples of infected humanized mice not linked to CRISPR-Cas9. Sequences were aligned to HIV-1_{NL4-3} sequence as a reference. The positions and nucleotide compositions of targets for gRNAs LTR1 and GagD are shown in green, PAMs in red. The sequencing data revealed lack of CRISPR-Cas9 specific cleavage (3 nucleotides from PAM) at the target sites LTR 1 (5'LTR in a for spleen lane

3 and 7, 3'LTR for kidney in c, lane 11 and in d for lung lane 4) or GagD (in c for kidney lane 7 and in e for liver and f for brain, lanes 2 and 16). Partial 3'LTR sequence was obtained for spleen lane 16 (b). Sanger sequencing results are included in FASTA format in the Source Data file for Fig. 6bc.

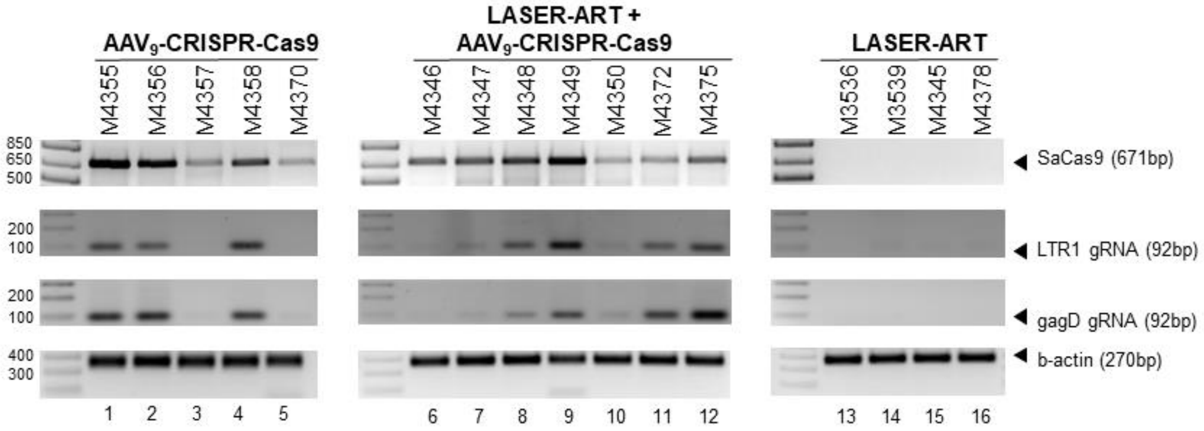

Supplementary Figure 8.

Cas9/gRNA expression in spleens of LASER ART and CRISPR-Cas9 treated and infected humanized mice. Reverse transcription- PCR analysis of RNA extracted from spleens of treated animals to represent SaCas9 mRNA (top panels), single guide RNAs: LTR 1 (second row panels) and GagD (third row panels), and a control beta-actin mRNA (bottom panels) were detected using primer sets specific to each target.

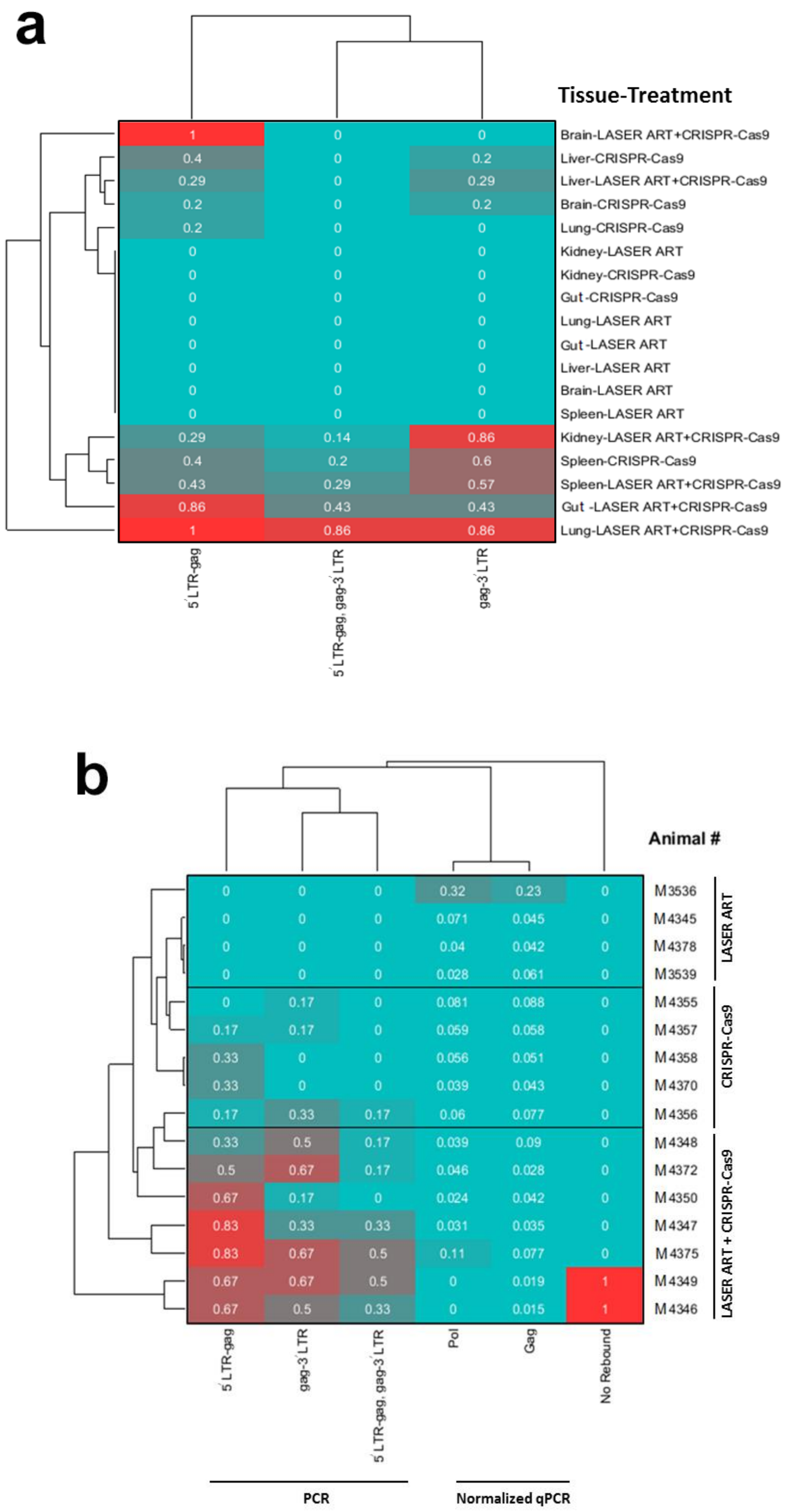
a
Tissue-Treatment
Brain-LASER ART+CRISPR-Cas9
Liver-CRISPR-Cas9
Liver-LASER ART+CRISPR-Cas9
Brain-CRISPR-Cas9
Lung-CRISPR-Cas9
Kidney-LASER ART
Kidney-CRISPR-Cas9
Gut-CRISPR-Cas9
Lung-LASER ART
Gut-LASER ART
Liver-LASER ART
Brain-LASER ART
Spleen-LASER ART
Kidney-LASER ART+CRISPR-Cas9
Spleen-CRISPR-Cas9
Spleen-LASER ART+CRISPR-Cas9
Gut-LASER ART+CRISPR-Cas9
Lung-LASER ART+CRISPR-Cas9
5′LTR-gag
5′LTR-gag, gag-3′LTR
gag-3′LTR
b
Animal #
M3536
M4345
M4378
M3539
M4355
M4357
M4358
M4370
M4356
M4348
M4372
M4350
M4347
M4375
M4349
M4346
LASER ART
CRISPR-Cas9
LASER ART + CRISPR-Cas9
5′LTR-gag
gag-3′LTR
5′LTR-gag, gag-3′LTR
Pol
Gag
No Rebound
PCR
Normalized qPCR

c

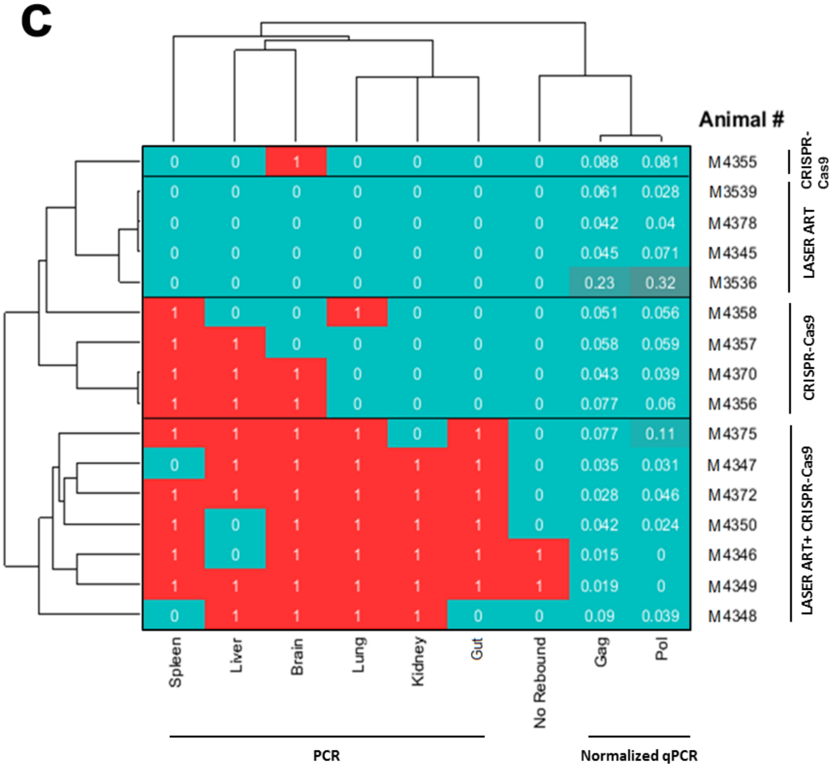

Supplementary Figure 9.

Hierarchical clustering of truncation efficiencies across animals, treatments, tissues and HIV-1 gene segments. Probabilities are shown with numbers and heat-map intensities. Similar groups are clustered together. Dendrograms indicate the hierarchy of clusters for each axis. (a) Clustering of truncation efficiencies of different HIV-1 segments in different tissues under LASER ART, CRISPR-Cas9, and LASER ART plus CRISPR-Cas9 treatments. The clustering reveals the most similarity between LASER ART plus CRISPR-Cas9-mediated editing in gut, spleen and lung. (b) Clustering of truncation efficiencies of different HIV-1 segments and qPCR data in different animals under LASER ART, CRISPR-Cas9, and LASER ART plus CRISPR-Cas9 treatments. The clustering scheme recognized similarity patterns and grouped the animals with similar treatments under the same clusters. (c) Clustering of truncation efficiencies in different tissues of the animals under the aforementioned treatments. Note that the animals with no rebound (treated with both LASER ART and CRISPR-Cas9 where virus was eliminated) exhibit similar patterns in excision probabilities across different HIV-1 segments and across different tissues. These analyses are later used in drawing the significance levels of combined treatment in viral genome eradication compared to the control groups. S1 refers to 5' LTR-Gag and S2 refers to Gag-3' LTR of the HIV-1 gene, respectively.

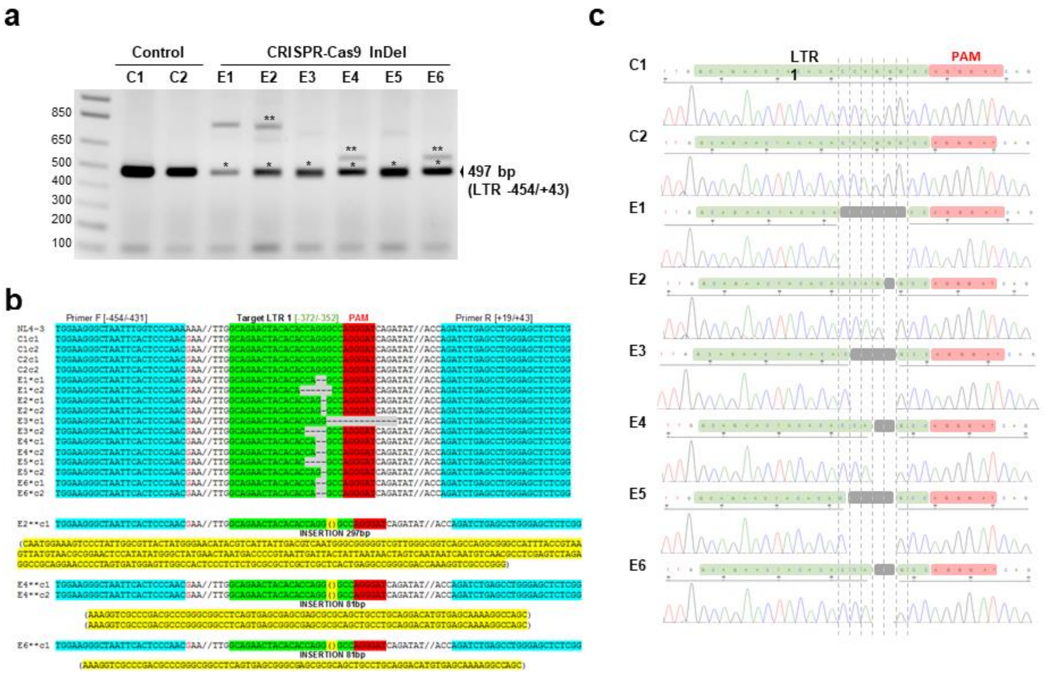

Supplementary Figure 10.

Off target cell model effects of CRISPR-Cas9. (a) Off target analysis of genomic DNA obtained from TZM-bl single cell clones: two controls (C1-2) and six Cas9/gRNA LTR 1 + GagD treated (E1-6). The presence of full length LTR -454/+43 (497bp) was examined. Amplicons containing CRISPR-Cas9 specific InDel mutations at the LTR 1 target site in integrated HIV-1 LTR sequence are pointed by asterisks. Single asterisks (*) indicate deletions, double asterisks (**) indicate insertions. (b) Alignment of a representative Sanger sequencing results of HIV-1 LTR specific amplicons. The positions and nucleotide compositions of target for gRNA LTR1 is shown in green, PAM in red, sequence deletions in grey, sequence insertions in yellow, and PCR primers in blue. (c) Representative Sanger sequencing tracing of LTR 1 region of HIV-1 LTRs obtained for each single cell clone. The positions and nucleotide compositions of target for gRNAs LTR1 is shown in green, PAM in red, sequence deletions in grey. Sanger sequencing results are included in FASTA format in the Source Data file.

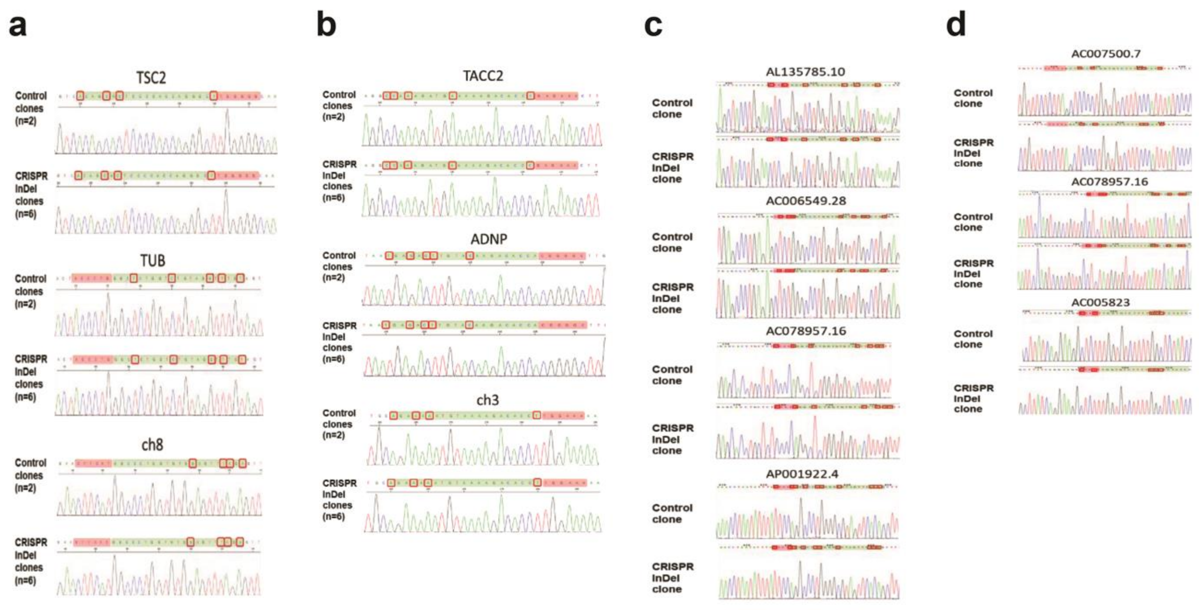

Supplementary Figure 11.

Representative Sanger sequencing tracing of predicted three off target regions of gRNAs LTR1 and GagD obtained for each single cell clone. Nucleotides comparing to target sequences. Top three of the LTR1 and GagD off-target sites locate at functional genes in (a) and (b). The off-target sites located at non-functional sequences are shown in (c) and (d), respectively. Sanger sequencing results are included in FASTA format in the Source Data file.

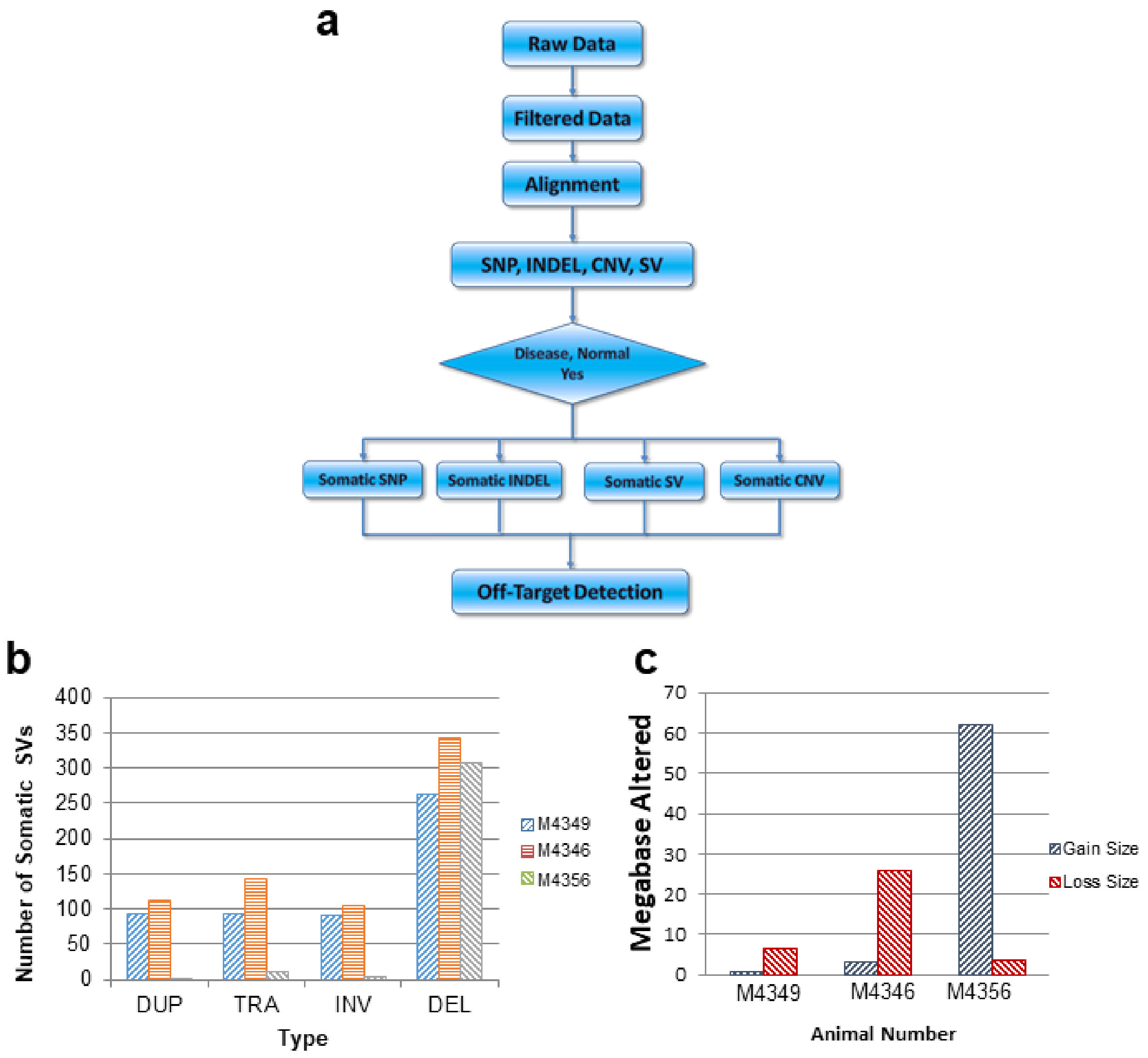

Supplementary Figure 12.

Appearance of somatic mutations in humanized mice. (a) Sequence of NGS data analysis steps used for off target detection. (b) Number of different types of somatic structural variations (SV) in each sample. Abbreviations: TRA: (Translocation) the number of translocations, INV: (Inversions) the number of inversions, DEL: (Deletion) the number of deletions, DUP: (Tandem duplication) the number of tandem duplications. (c) The size of genomic regions affected by somatic CNVs in each sample. After identification of all somatic alterations (detected with respect to the human reference genome) and potential off-targets and comparing them using blast, it has been concluded that none of the predicted off-targets coincide with the identified variations including SNPs, SVs, CNVs and InDel mutations, rejecting the occurrence of CRISPR-Cas9 off-target cleavage.

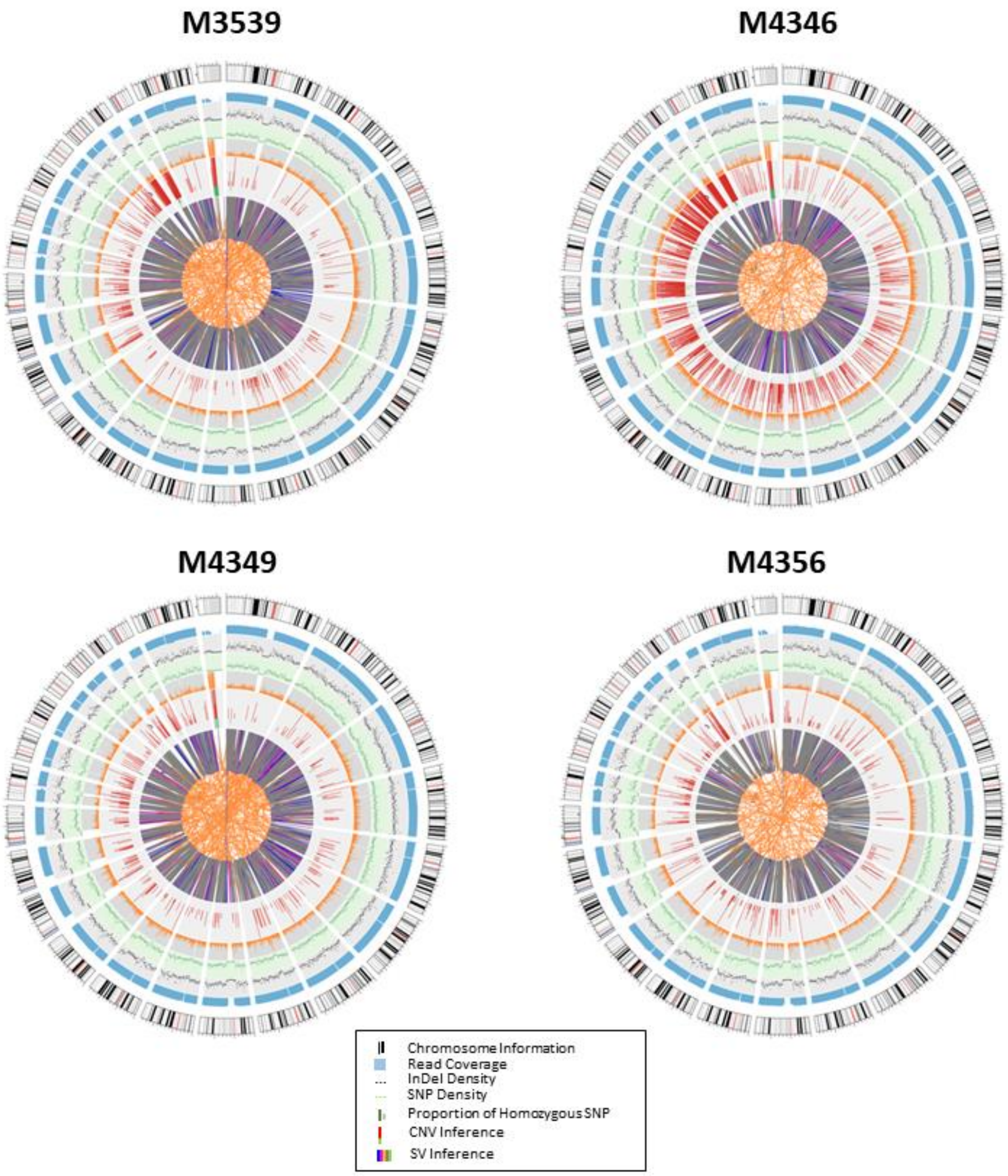

Supplementary Figure 13.

Circos diagrams of the infected humanized mice (a), M3539 (LASER ART), (b) M4346 and, (c) M4349 (LASER ART and CRISPR-Cas9), and (d) M4356 (CRISPR-Cas9). The diagrams consist of seven outer to inner rings: (1) The outer circle (the first circle) is chromosome information. (2) The second ring represents the read coverage in histogram style. A histogram is the average coverage of a 0.5Mbp region. (3) The third ring represents InDel density in scatter style. A black dot is calculated as InDel number in a range of 1Mbp. (4) The fourth ring represents SNP density in scatter style. A green dot is calculated as SNP number in a range of 1Mbp. (5)

The fifth ring represents the proportion of homozygous SNP (orange) and heterozygous SNP (grey) in histogram style. A histogram is calculated from a 1Mbp region. (6) The sixth ring represents the CNV inference. Red means gain, and green means loss. (7) The most central ring represents the SV inference in exonic and splicing regions. TRA (orange), INS (green), DEL (grey), DUP (pink) and INV (blue). The genetic variations of each treatment have been detected after comparison of the NGS data with the human reference genome).

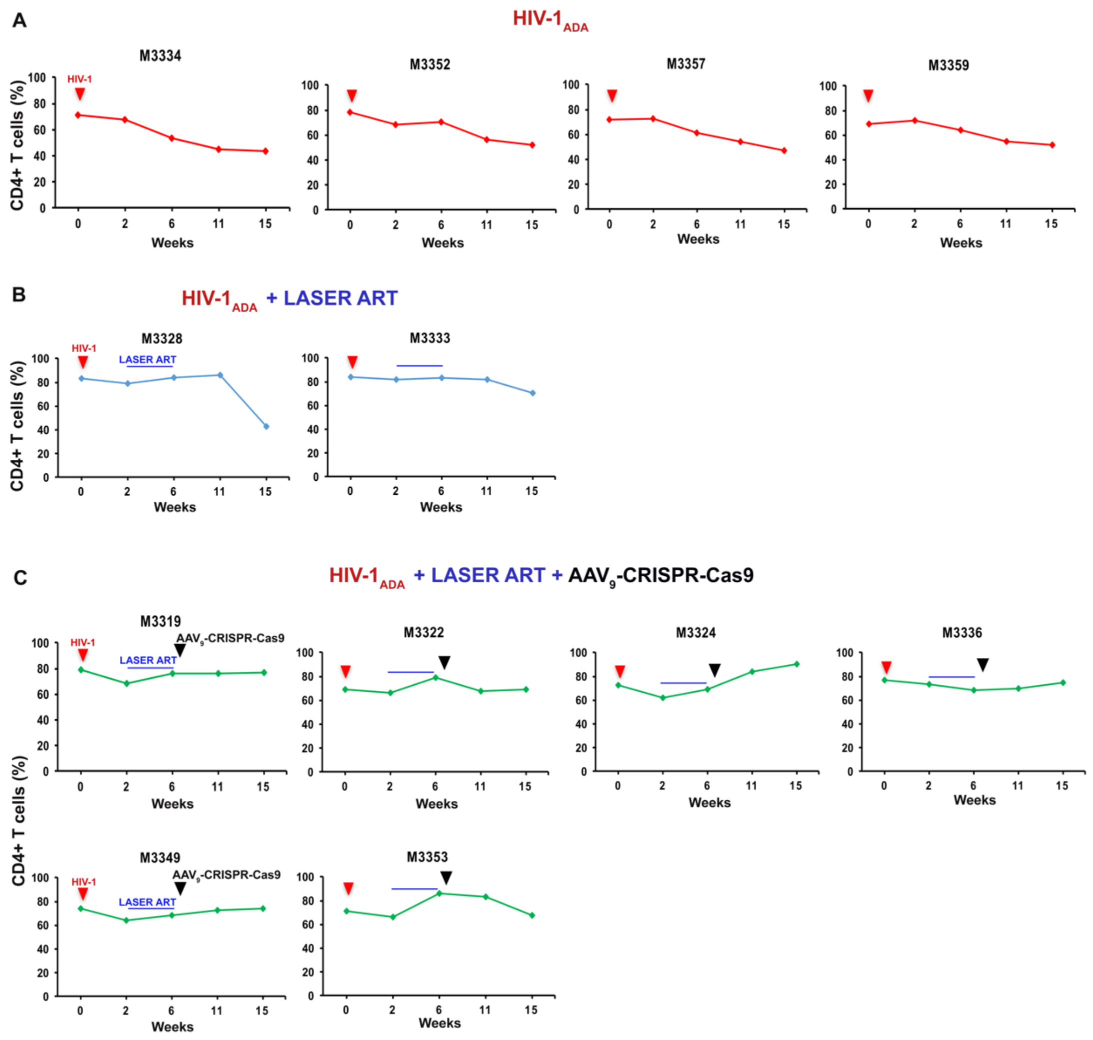

Supplementary Figure 14.

Flow cytometric evaluations of human CD4+ T cells in humanized mice.

Peripheral blood of CD34+ NSG-humanized mice was assayed before (0) and 2, 6, 11, and 15 weeks post-infection with HIV-1_{ADA} for human CD4+ cells from CD3+ gated populations. These experiments assessed CD4+ T cells patterns throughout the course of the study. (a) The percentage of human CD4+ T cells followed a decreasing pattern in all four mice in the HIV-1_{ADA} infected group. (b) CD4+ T cell profile of HIV-1_{ADA} and LASER ART animals. (c) CD4+ T cell profile of HIV-1_{ADA} and LASER ART and AAV_9-CRISPR-Cas9 animals (n=6). A decline in percentage of CD4+ T cells was observed two weeks after infection in all 6 mice, after which the LASER ART treatment was followed for four more weeks (6 weeks post infection), followed by AAV_9-CRISPR-Cas9 injection. The mice were then followed as per the study design for nine more weeks where they were found to have restoration of CD4+ T cells.

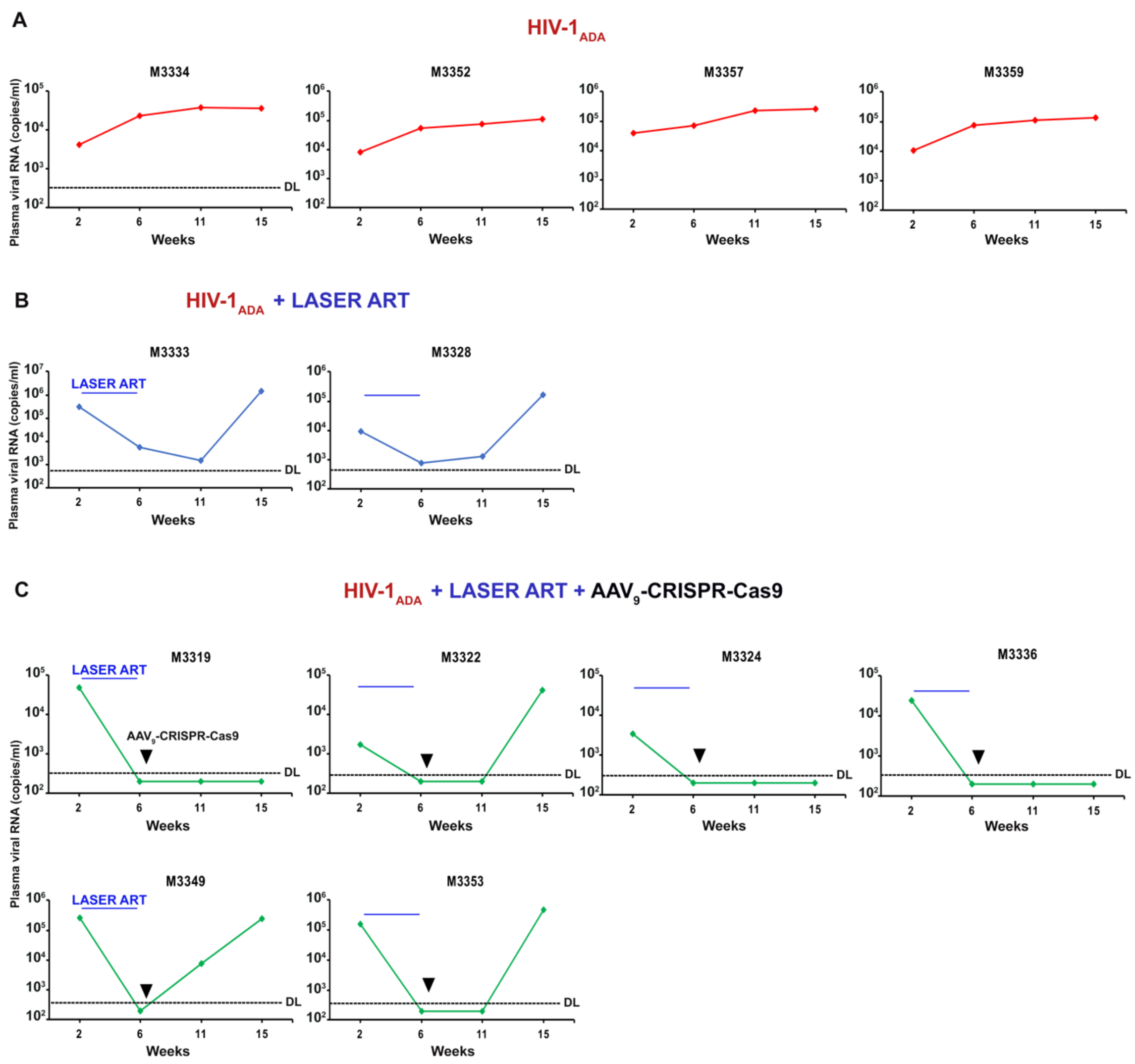

Supplementary Figure 15.

Viral load measurements in humanized mice.

Plasma viral load of CD34-NSG-humanized mice was assayed 2, 6, 11, and 15 weeksafter HIV-1 infection for the presence of HIV-1 RNA using the Roche COBAS Ampliprep-Taqman-48 V2.0 kit. The sensitivity of the assay was adjusted to 200 copies/ml based on sample dilution. These experiments were performed to assess the level of viral infection throughout the study. (a) Viral load of all four HIV-1$_{ADA}$ infected animals were examined. (b) Viral load profile of HIV-1 and LASER ART animals. (c) Viral load profile of all HIV-1$_{ADA}$ and LASER ART and CRISPR-Cas9 animals (n=6) are shown. We observed a rebound of viral RNA at the study end in three out of six dual treated animals, which corresponds to nine weeks after therapy interruption. No viral rebound was observed in three dual treated animals (M3319, M3324 and M3336).

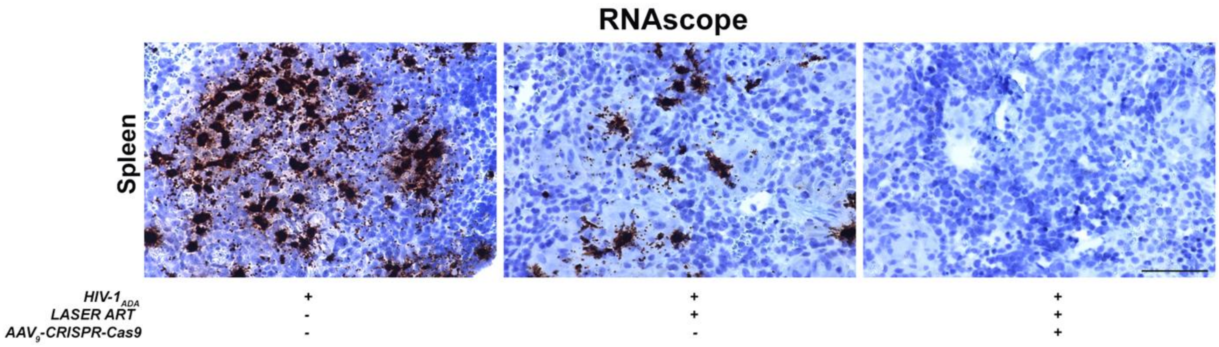

Supplementary Figure 16.

RNAscope analyses of HIV-1$_{ADA}$ infected and treated humanized mice. RNAscope in situ hybridization was used to detect viral RNA in 5-μm thick sections of spleens (single brown dots or cluster of dots). The assays used antisense probeV-HIV-1-Clade-B targeting 854-8291 base pairs of the HIV-1 genome. Viral RNA was detected in other 2 groups of humanized mice spleen (HIV-1$_{ADA}$ infected, and infected and LASER ART treated). Mouse M3319 which received LASER ART and CRISPR-Cas9, where virus was not detected. The photomicrographs are representative images from each group. Human peptidyl Isomerase B was used as a positive control for all tissues analyzed. Scale bar, 40μm.

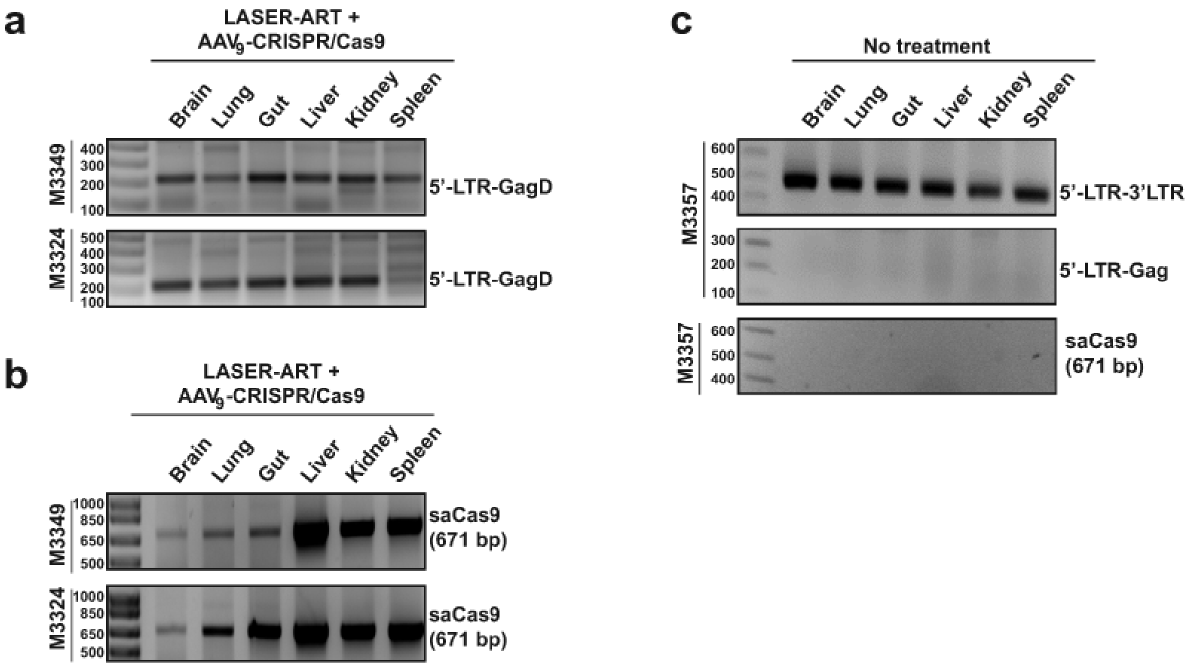

Supplementary Figure 17.

Excision of HIV-1 proviral DNA by CRISPR-Cas9 in HIV_{ADA}-infected humanized mice. A much shorter fragment (193 bp) of excised HIV proviral DNA from the 5'LTR to Gag region was amplified by nested-PCR in total genomic DNA extracted from various tissues of humanized mice (M3324 and M3349) (a) along with the presence of SaCas9 DNA in each tissue (b). HIV excision was not detected in the humanized mouse treated with LASER ART only (M3357) even though a full length of HIV-1 LTR could be amplified abundantly to reveal the existence of HIV proviral DNA (c). All 5'-LTR-GagD truncated amplicons were verified by Sanger sequencing and sequences are included in FASTA format in the Source Data file.

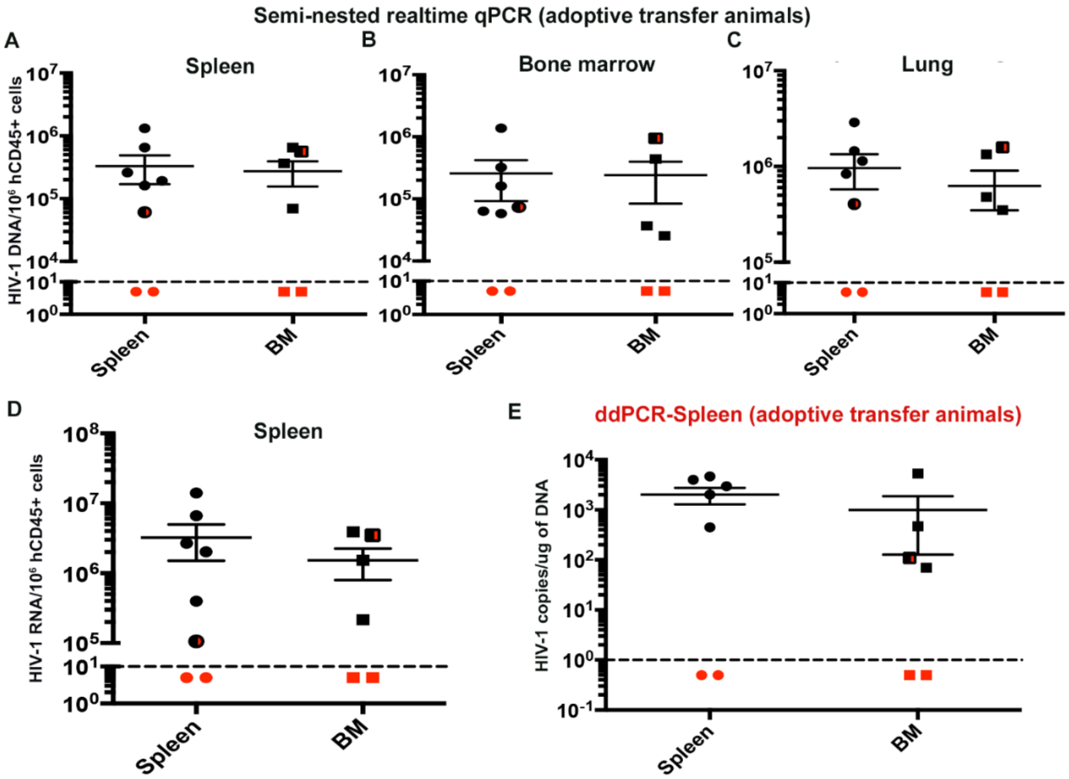

Supplementary Figure 18.

Detection of HIV-1$_{ADA}$ DNA and RNA in spleen tissues in adoptively transferred humanized mice. Splenocytes and bone marrow (BM) cells were isolated from HIV-1 infected mice with or without prior LASER ART and or CRISPR-Cas9 treatments. These were for adoptive transfers into new CD34+ NSG-humanized mice. The intent was to perform cross disciplinary viral amplification from known infectious cell reservoirs. (a, b and c) HIV-1 DNA and (d) RNA analyses using ultrasensitive semi-nested real-time qPCR assays from spleen, bone marrow and lung tissues of adoptively transferred humanized mice. The data are expressed as total HIV-1 DNA or RNA copies/10^6 human CD45+ cells. Four animals (splenocyte and BM cells isolated and adoptively transferred from M3319 and M3336) (shown by red circles and squares below dotted line), showed no viral recovery. The above data was further confirmed using ultrasensitive ddPCR assay where the same adoptively transferred recipient animals showed no HIV-1 (e) indicating complete elimination of virus. Virus was recovered from HIV-1$_{ADA}$ infected mice with or without LASER ART treatment from spleen tissues. These results provide evidence of viral elimination in the two mice (M3319 and M3336) that showed no evidence for rebound after LASER ART cessation and had no viral DNA after CRISPR-Cas9 treatment.

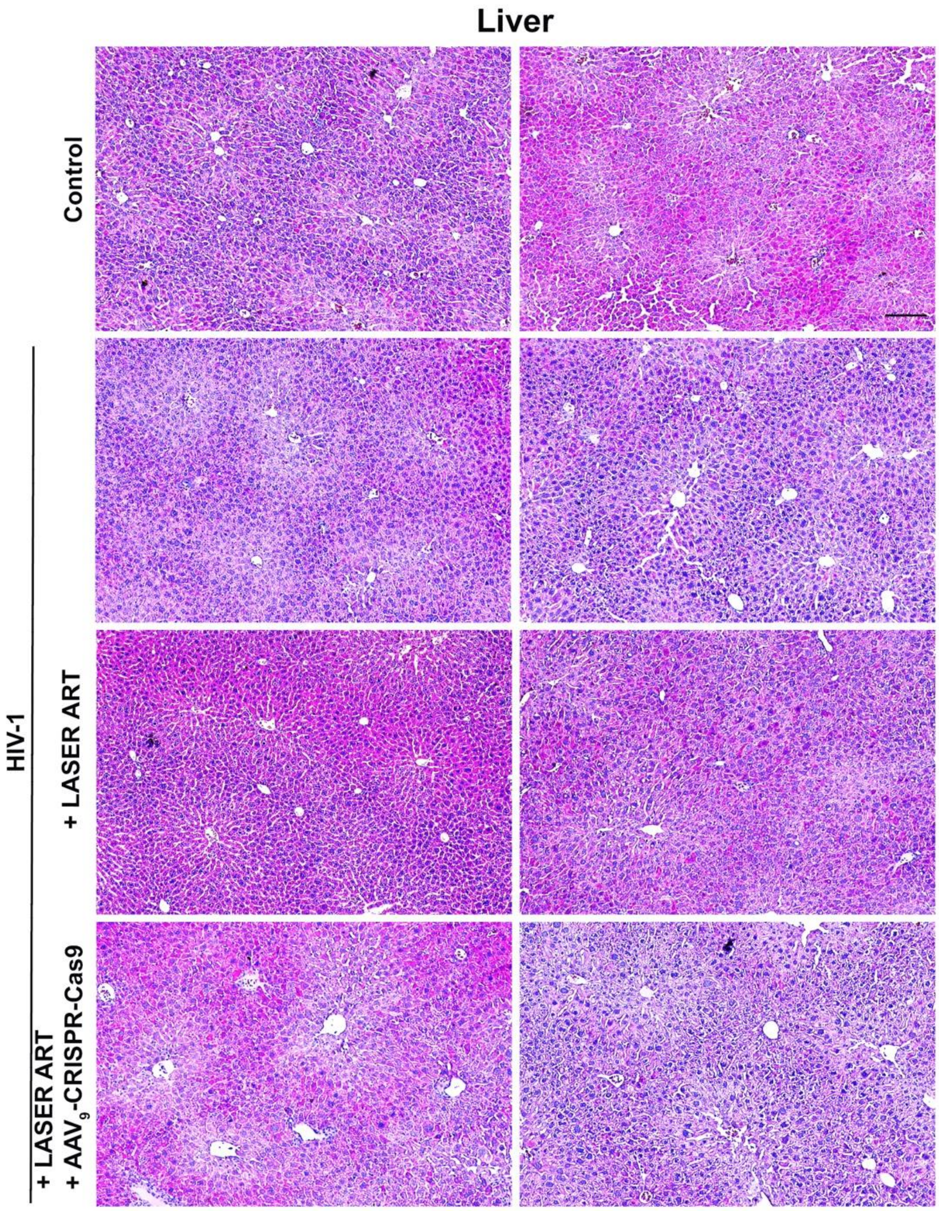

Supplementary Figure 19.

Liver tissue histology in treated humanized mice. Hematoxylin and eosin staining of representative sections from liver tissues in uninfected, HIV-1$_{ADA}$-infected, infected and LASER ART treated and dual treated (LASER ART and CRISPR-Cas9) humanized mice at the endpoint of the study. We did not observe any tissue pathology in LASER ART alone or dual treatment groups. Scale bar, 10μm.

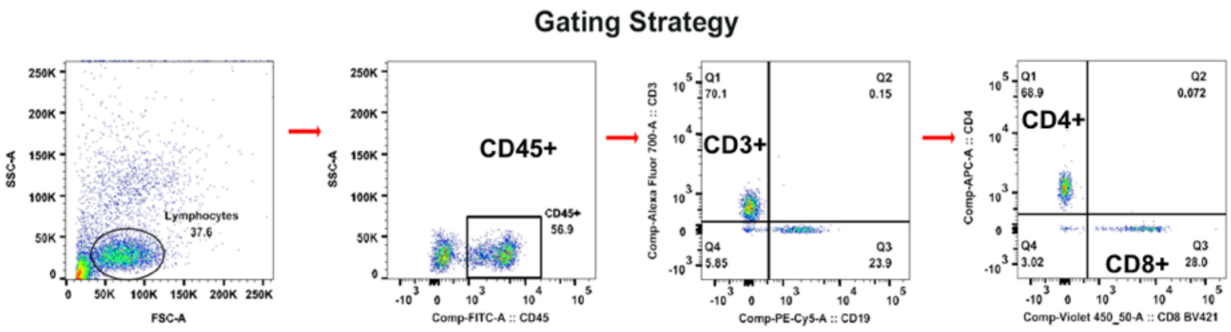

Supplementary Figure 20.

Flow cytometric analysis of peripheral blood mononuclear cells. Blood cells were gated for mononuclear cells and lymphocytes using forward and side scattered panels (FSC and SSC). From the gated lymphocyte population, human CD45+ cells were re-gated in side-scatter panel. Gated human CD45+ mononuclear cells were assessed for expression of human CD3 (T cells) and CD19 (B cells). CD3+ T cells were further gated to assess the expression of CD4 and CD8 cells. This pattern of gating was used for Figures 1, 2b, 3, 4 a-b and 7b and supplementary figure 14.

Supplementary Table 1. **Polymerase Chain Reaction (PCR) primers and probes**

	primer	sequence
Standard PCRs		
5'LTR-Gag	1st round LTR F	5'-AATTGCGGCCGCTGGAAGGGCTAATTTGGTCCC-3'
	1st round Gag R	5'-TGTCACTTCCCCTTGGTTCTCTC-3'
	nested 5'LTR F	5'-AAAAGAATTCGTGGATCTACCACACACAAGGC-3'
	nested Gag R	5'-AAAAGGATCCACCATTTGCCCCTGGAGGTT-3
Gag-3'LTR	1st round Gag F	5'- GAAAGCGAAAGTAAAGCCAGAGGAGAT-3
	1st round LTR R	5'-ACACAACAGACGGGCACACACTACTT -3'
	nested Gag F	5'AAAAGAATTCGACAGCTACAACCATCCCTTCAGACAG-3'
	nested 3'LTR R	5'-AAAAGGATCCAGCAGTGGGTTCCCTAGTTAGCCAG-3'
LTRs	1st round LTR -413/S	5'-TTGGCAGAACTACACACCAGGG -3'
	1st round LTR +43/AS	5'-CCGAGAGCTCCCAGGCTCAGATCT-3'
	nested LTR -374/S	5'-TTAGCAGAACTACACACCAGGGCC-3'
	nested LTR -19/AS	5'-GCTGCTTATATGTAGCATCTGAG-3'
Hs beta-globin	Hs b-globin F	5'-CCCTTGGACCCAGAGGTTCT-3'
	Hs b-globin R	5'-CGAGCACTTTCTTGCCATGA-3'
Mm beta-globin	Mm b-globin F	5'-CCCTTGGACCCAGCGGTACT-3'
	Mm b-globin R	5'-GTTATCACCTTCTTGCCATG-3'
Taqman qPCRs		
LTRs	HIV-1LTR-338/F	5'-CCACTGACCTTTGGATGGTGCT-3'
	HIV-1LTR-109/R	5'-CCCAGCGGAAAGTCCCTTGT-3'
	HIV-1LTR-163/probe	5'-FAM-ACTCCGGATGCAGCTCTCGGGCCA-ZEN-IowaBlackFQ-3'
Pol	HIV-1 pol/int F	5'-TCCAGCAGAGACAGGGCAAG-3'
	HIV-1 pol/int R	5'-TGCCAAATTCCTGCTTGATCCC-3'
	HIV-1 pol/int probe	5'-HEX-CGCCCACCAACAGGCGGCCTTAACTG-ZEN-IowaBlackFQ-3'
Gag	HIV-1 Gag F	5'-TCAGCCCAGAAGTAATACCCATGT-3'
	HIV-1 Gag R	5' -CACTGTGTTTAGCATGGTGTTT - 3'
	HIV-1 Gag Probe	5'-FAM-ATTATCAGAAGGAGCCACCCCACAAGA-ZEN-IowaBlackFQ-3'
Env	HIV-1 Env F	5'- TCCTTGGGATGTTGATGATCT-3'
	HIV-1 Env R	5'- TGGCCCAAACATTATGTACC-3'
	HIV-1 Env Probe	5'-FAM-TGGTGGTTGCTTCTTTCCACACA-ZEN-IowaBlackFQ-3'
Reference	Hs b-globin F	5'-CCCTTGGACCCAGAGGTTCT-3'
	Hs b-globin R	5'-CGAGCACTTTCTTGCCATGA-3'
	Hs b-globin probe:	5'-FAM-GCGAGCATCTGTCCACTCCTGATGCTGTTATGGGCGCTCGC-ZEN-IowaBlackFQ-3'
	Hs b-actin F	5'-TGGACTTCGAGCAAGAGATG-3'
	Hs b-actin R	5'-GAAGGAAGGCTGGAAGAGTG-3'
	Hs b-actin probe	5'-FAM-CGGCTGCTTCCAGCTCCTCC-ZEN-IowaBlackFQ-3'
	Mm b-actin F	5'-TCGCTCTCTCGTGGCTAGTA-3'
	Mm b-actin R	5'-TAGGCGTAAAGTTGGCTGTG-3'
	Mm b-actin probe	5'-FAM-CGCCCATGGTGTCCGTTCTG-ZEN-IowaBlackFQ-3'
RT-PCRs		
	LTR1/F	5'-GCAGAACTACACACCAGGGCC-3'
	GagD/F	5'-GGATAGATGTAAAAGACACCA-3'
	pX601gRNAscaffold/R	5'-CGCCAACAAGTTGACGAGAT-3'
	SaCas9/263/F	5'-TCGACTACAACCTGCTGACC-3'
	SaCas9/R	5'-GGTGGGCTTCTTCTGCTT-3'

	b- actin S	5'-CTACAATGAGCTGCGTGTGGC-3'
	b-actin AS	5'-CAGGTCCAGACGCAGGATGGC-3'
In vitro OFF target analysis		
LTR 1 OFF targets	LTR1OFFch8/F	5'-GAGTGACCTTCCCAAATTGC-3'
	LTR1OFFch8/R	5'-ATGGTGAGGTGAGGGATGAG-3'
	TSC2/35001F	5'-CAGACTCTGATGGGTGGCAG-3'
	TSC2/35398R	5'-GCTAAGGAGAGAGGGTGGGA-3'
	TUB/66607F	5'-CCAAGTGGCCCTCAGATTACA-3'
	TUB/67015R	5'-TCATTCACCCCAAATCCTACGG-3'
	SLC41A2/F	5'-GAGCAGGAAGGAGCTGTGG-3'
	SLC41A2/R	5'-GAGACTTCCCCTCCATGGTC-3'
	AL135785.10/F	5'-ATCTGTTGTGTGTCTATGAGCCCTG-3'
	AL135785.10/R	5'-AACTTGTTCATGGCTCACTGTTTG-3'
	AC006549.28/F	5'-CCTCACACTTGCCCTATCCACAG-3'
	AC006549.28/R	5'-GGTGTGTTCGCCCATCAGG-3'
	AP001922.4/F	5'-CCCATCCAACTCCGTATCTCAAG-3'
	AP001922.4/R	5'-GGTGTTTTCAAGGGCTTTGTTTG-3'
	AL1159977.10/F	5'-TAACACGAATCACTAGGCAACAGGAA-3'
	AL1159977.10/F	5'-CAAAGAAGAACACAGACTCCAGCATC-3'
Gag D OFF targets	GagDOFFch3/F	5'-CATTAACCACCTGGGGAACA-3'
	GagDOFFch3/R	5'-TCTCAGACCCAGGAATGTCA-3'
	TACC2/392F	5'-GAGGACTCTCCAGCCAAAGG-3'
	TACC2/782R	5'-GAGCTGGGGGTCTTAGAGGA-3'
	ADNP/41574F	5'-TGCACCAGCCAAAACTTAGGA-3'
	ADNP/41996R	5'-TCTAATTAGGTGGCAGCACGTT-3'
	AC007500.7/F	5'-CGAATGCAGAAGAAAATGAGACAGTG-3'
	AC007500.7/R	5'-TCACCAGGCACGGGATCTCTC-3'
	AC078957.16/F	5'-ATCAAATGCTTCACTGCCCTGTTA-3'
	AC078957.16/R	5'-CTGTAAGCCGTTCCTTTCTGGGT-3'
	AC005823.1/F	5'-CAGCATCTAACACCAAGCCAAGG-3'
	AC005823.1/R	5'-GGGAGAAGGAGCCAACCAAGTC-3'
Cloning gRNAs protospacers into pX601 AAV vector		
Cloning single gRNAs into pX601 AAV vector	LTR1 gRNA top	5'-CACCGCAGAACTACACACCAGGGCC-3'
	LTR1 gRNA bottom	5'-AAACGGCCCTGGTGTGTAGTTCTGC-3'
	GagD gRNA top	5'-CACCGGATAGATGTAAAAGACACCA-3'
	GagD gRNA bottom	5'-AAACTGGTGTCTTTTACATCTATCC-3'
Multiplexing gRNA cassettes	InFusion/T795/F	5'- ATTACGCTTAAGAATTCCTAGAGC-3'
	InFusion/T796/R	5'-GGAAATAGGCCCTCAGACTAGGGGTTCCTGCGGCCGCAAA-3'

Supplementary Table 2. Potential off target sites in human genome for HIV-1 target LTR 1 and GagD

HIV-1 LTR 1 target (+ strand)

GCAGAACTACACACCAGGGCCAGGGAT

Sequence	PAM	Score	Gene	Chromosome	Strand	Position	Mismatches	On-target	Verified
TCTAAACTCCACACCAGGGCC	ATGAA	2.6		chr8:+22915337	1	22915337	4	FALSE	*
TCAGATCTCCACACCAGAGCC	ACGAG	1.3		chr9:+38360364	1	38360364	4	FALSE	*
ACAGGCCAACCCACCAGGGCC	CAGAG	0.9		chr22:-20136959	-1	20136959	5	FALSE	*
GTAGGACTACGCACCAGGGCA	AAGAG	0.9		chr8:-92102695	-1	92102695	4	FALSE	*
ACAAAAGTACACACCAGAGCC	TGGGG	0.8		chr11:+75625035	1	75625035	4	FALSE	*
TGTGAACTACGCCCCAGGGCC	TGGAA	0.8		chr13:-27341725	-1	27341725	5	FALSE	
ACAGAGCTGAGCACCAGGGCC	CAGGG	0.8		chr10:+124217737	1	1.24E+08	5	FALSE	
CCAGTTCTCCACCCCAGGGCC	ATGGA	0.8		chr15:+28948038	1	28948038	5	FALSE	
CCAGAGCTGCTTACCAGGGCC	ATGGA	0.7		chr1:-47650696	-1	47650696	5	FALSE	
ACAGCACTCCCCACCAGGGCT	TGGGG	0.7	TSC2	chr16:-2082981	-1	2082981	5	FALSE	*
ACAGAACGTCACACCAGGGTC	AGGAG	0.7		chr7:+26573832	1	26573832	4	FALSE	
ACAAAACTAGACAGCAGGGCC	AGGAG	0.7		chr19:-54347353	-1	54347353	4	FALSE	
TGAGCACTTCACAGCAGGGCC	GGGAA	0.7		chr2:+43112424	1	43112424	5	FALSE	
GCAGCACTACACATCAGGGCT	AAGAA	0.7		chr16:-60058984	-1	60058984	3	FALSE	
CCGCAACTCCACAGCAGGGCC	AGGGA	0.7		chr15:+80851232	1	80851232	5	FALSE	
CTAGAGGAACACACCAGGGCC	TGGGA	0.6		chrX:-103784275	-1	1.04E+08	5	FALSE	
ACAGCCCCAGACACCAGGGCC	TGGAG	0.6		chr15:-57542258	-1	57542258	5	FALSE	
CCAGGTCTACCCAGCAGGGCC	AGGAG	0.6		chr11:+121718784	1	1.22E+08	5	FALSE	
ACAGGAGGGCACACCAGGGCC	CAGGA	0.6		chr13:-47252260	-1	47252260	5	FALSE	
ACAGAAATAAACACCAGGGCT	TCGGG	0.6		chr2:-12064330	-1	12064330	4	FALSE	
GCAGAACTGCAGACCAGGGGC	TGGGG	0.6		chr11:-76235199	-1	76235199	3	FALSE	
CCAGAGCACCAAACCAGGGCC	CAGGA	0.5		chr2:-238434996	-1	2.38E+08	5	FALSE	
GCAGAGCTCCCCACCAGGGGC	AGGGA	0.5		chr2:-127586882	-1	1.28E+08	4	FALSE	
ACAGGCCCACACTCCAGGGCC	CAGAA	0.5		chr5:-134248836	-1	1.34E+08	5	FALSE	
GCAGTGCCACACTCCAGGGCC	TTGGG	0.5		chr19:-11048922	-1	11048922	4	FALSE	
GCAGGAGTAGGCACCAGGGCC	CTGAG	0.5		chr1:-41442886	-1	41442886	4	FALSE	
GCAGCACCACACACCAGGCCC	AGGAG	0.5		chr14:-96723779	-1	96723779	3	FALSE	
GCAGAGCTAGCCACCAGGGCT	TAGGA	0.4		chr6:-137609940	-1	1.38E+08	4	FALSE	
GCAGAGCTCCAGCCCAGGGCC	TGGGG	0.4		chr22:+49956739	1	49956739	4	FALSE	
GGGGAAATACACATCAGGGCC	AGGAA	0.4		chr20:-43964342	-1	43964342	4	FALSE	
AGAGAATTTCACAACAGGGCC	CTGAA	0.4		chr3:-189039375	-1	1.89E+08	5	FALSE	
ACAAACCTACAGACCAGAGCC	CAGGG	0.4	TUB	chr11:-8105357	-1	8105357	5	FALSE	*
CATGAGCTACACACCAGGACC	AGGAG	0.4		chr7:+47512275	1	47512275	5	FALSE	
GAAAAACTACAGACCAGGGAC	AAGGG	0.4		chr6:-68746690	-1	68746690	4	FALSE	

CCAGAACTCAGCCCCAGGGCC	CTGGG	0.4		chr5:-137113375	-1	1.37E+08	5	FALSE	
GCTGGCCTACACACCAGGCCC	AGGGG	0.4		chr3:+38015758	1	38015758	4	FALSE	
CCTGAACCACACCCCAGGGCT	CAGGG	0.3		chr2:-128441625	-1	1.28E+08	5	FALSE	
GCAGAACACCAAGCCAGGGCC	AGGAA	0.3		chr10:-95607490	-1	95607490	4	FALSE	
GAATAGCTACACACTAGGGCC	ATGGA	0.3		chr2:-69175215	-1	69175215	4	FALSE	
GAAGAACCACAAAACAGGGCC	CAGAA	0.3		chrX:+43803871	1	43803871	4	FALSE	
ATAGTACTACACTCCTGGGCC	TCGAG	0.3		chr5:+5184057	1	5184057	5	FALSE	
GAAGAACAACACAGCAGGGCA	GAGAG	0.2	TBC1D19	chr4:+26576719	1	26576719	4	FALSE	
CCAGAAACACCCACCAGTGCC	CGGGA	0.2		chr19:+15265258	1	15265258	5	FALSE	
CCAGAGCTGCAGACCCGGGCC	CCGGG	0.2		chr9:-133679870	-1	1.34E+08	5	FALSE	
CCAGACCGAGACACCAGGGGC	GGGGG	0.2	SLC41A2	chr12:+104958165	1	1.05E+08	5	FALSE	*
CCAGATCTAGACTCCAGGGCA	GTGAG	0.2		chr1:-203423414	-1	2.03E+08	5	FALSE	
TCAGAGCTAGACTCCAGGGCT	GGGGG	0.2		chr19:-48393411	-1	48393411	5	FALSE	
GGAGAACTTAACACCAGGTCC	CTGGG	0.2		chr22:-41003477	-1	41003477	4	FALSE	
CCAGCACCACAGAGCAGGGCC	TGGGA	0.2		chr11:+319276	1	319276	5	FALSE	
CCAGCACCACAGAGCAGGGCC	TGGGA	0.2		chr11:-310505	-1	310505	5	FALSE	

HIV-1 Gag D target (+strand)

GGATAGATGTAAAAGACACCAAGGAAG

Sequence	PAM	Score	Gene	Chromosome	Strand	Position	Mismatches	On-target	
AGAAAAATGTAAAAGACACCT	TGGAA	1.7		chr3:-144746442	-1	1.45E+08	4	FALSE	*
TTATACATTTGAAAGACACCA	AAGAA	1.5		chr1:-194738918	-1	1.95E+08	5	FALSE	
GGATAAATGGGAAAGACACCA	GGGGA	1.5		chr16:-48814775	-1	48814775	3	FALSE	*
TCTTAGACTTAAAAGACACCA	TTGAA	1		chr15:-33069866	-1	33069866	5	FALSE	
ACATTGAATTAAAAGACACCA	TAGAG	1		chrX:-32002168	-1	32002168	5	FALSE	*
GGATAGAGCCAAAAGACACCA	AAGAG	1		chr17:-51350241	-1	51350241	3	FALSE	*
AAATAGCTCTTAAAGACACCA	GCGAA	0.9		chr2:+173764948	1	1.74E+08	5	FALSE	
AGATCAATGTAAAAGTCACCA	TCGAA	0.9		chr6:-144452168	-1	1.44E+08	4	FALSE	
TTTTAGATGTAAAAGACATCA	GGGAG	0.8		chr3:+187644948	1	1.88E+08	4	FALSE	
TGATAAATGAAACAGACACCA	GAGGA	0.8		chr7:-141719859	-1	1.42E+08	4	FALSE	
GAAAAGATTTAAGAGACACCA	AAGAG	0.8		chr2:-213166139	-1	2.13E+08	4	FALSE	
AGGGAGATCTAAGAGACACCA	GAGAG	0.8		chr19:-29842353	-1	29842353	5	FALSE	
ATGCAGATGTAACAGACACCA	GGGAA	0.8		chr1:-226725698	-1	2.27E+08	5	FALSE	
GTATGGATGTTAAAGACTCCA	TTGAG	0.7		chr5:-142976527	-1	1.43E+08	4	FALSE	
CGGTAGATTTTAAAGACTCCA	AAGAG	0.7		chr9:-38648188	-1	38648188	5	FALSE	
AGAGAGATATTAAAGACCCCA	GTGAA	0.6		chr18:-43665283	-1	43665283	5	FALSE	
GGATAAATGTGAAAGACATCA	TAGAA	0.6		chr18:+51783716	1	51783716	3	FALSE	
AGAAGGAGGAAAAAGACACCA	GGGAG	0.6		chr2:+218083925	1	2.18E+08	5	FALSE	
TAATAGGTAGAAAAGACACCA	GTGAA	0.6		chr12:-126150002	-1	1.26E+08	5	FALSE	
CCAAAGATGAAAAAGACACCC	GAGAA	0.6	TACC2	chr10:+122211347	1	1.22E+08	5	FALSE	*

TTATAAATGCAAAAGACACCC	ATGAA	0.6		chr14:-46407726	-1	46407726	5	FALSE	
GGCTGGGTGAAAAAGACACCA	TGGAA	0.6		chr6:+66585737	1	66585737	4	FALSE	
GGACAGATGTGAAAGAGACCA	AAGGA	0.5		chr2:+224685483	1	2.25E+08	3	FALSE	
TGATGCAAGTAACAGACACCA	TGGGA	0.5		chr6:+107524588	1	1.08E+08	5	FALSE	
CAATAGTTGTTCAAGACACCA	GTGAA	0.5		chr6:-156459061	-1	1.56E+08	5	FALSE	
AGAAAGATACAGAAGACACCA	GGGAG	0.5		chr11:-75223569	-1	75223569	5	FALSE	
TGAGACTTGTACAAGACACCA	CGGGG	0.5	ADNP	chr20:-50889247	-1	50889247	5	FALSE	*
AGATTGTTGGTAAAGACACCA	CAGAG	0.5		chr7:-114527499	-1	1.15E+08	5	FALSE	
GGAAAGTTATAAAAGACACCG	GGGAA	0.5		chr7:+99103371	1	99103371	4	FALSE	
CCATTGATCTAAAAGTCACCA	CTGGA	0.5		chr3:-65736276	-1	65736276	5	FALSE	
AAATACCTGTAAGAGACACCA	CTGAG	0.5		chr3:-65976946	-1	65976946	5	FALSE	
TGGTAGATTTATAAGACACCG	TAGGG	0.5		chr10:-3117938	-1	3117938	5	FALSE	
GAATGGATGTGAAAGGCACCA	CTGAA	0.5		chr5:-79875682	-1	79875682	4	FALSE	
AAATAAATGTGAAAGTCACCA	CAGAA	0.5		chr8:-131973280	-1	1.32E+08	5	FALSE	
AGATGGATGGCATAGACACCA	CGGGG	0.4		chr3:+52369345	1	52369345	5	FALSE	
TGAAAGATCTTAAAGCCACCA	AAGGA	0.4		chr20:-24138791	-1	24138791	5	FALSE	
GAGTAGATCTAAAAGACAGCA	AGGAA	0.4		chr12:-62718609	-1	62718609	4	FALSE	
TCATATGTGTAAAAGACACAA	AGGAG	0.4		chr2:+3551648	1	3551648	5	FALSE	
GGTTAGCGGGAAAAGACACCA	CAGGG	0.4		chrX:-141696540	-1	1.42E+08	4	FALSE	
GGTTAGCGGGAAAAGACACCA	CAGGG	0.4		chrX:+141591639	1	1.42E+08	4	FALSE	
GGTTAGCGGGAAAAGACACCA	CAGGG	0.4		chrX:-141582808	-1	1.42E+08	4	FALSE	
GGTTAGCGGGAAAAGACACCA	CAGGG	0.4		chrX:-141240587	-1	1.41E+08	4	FALSE	
GGTTAGCGGGAAAAGACACCA	CAGGG	0.4		chrX:+141004580	1	1.41E+08	4	FALSE	
GGATTCATGCAAAAGACACTA	TAGGG	0.4		chr3:-85730118	-1	85730118	4	FALSE	
AGAAATATCTAAAAGACAACA	AAGAG	0.4		chr7:+122888645	1	1.23E+08	5	FALSE	
GGAAAGGAGCAAAAGACACCA	GAGGG	0.4		chr17:-81470363	-1	81470363	4	FALSE	
AGATTCATTTAAAAGACAACA	AAGAA	0.4		chr8:-109514887	-1	1.1E+08	5	FALSE	
AGAGATATGTATAAGACACAA	TAGGA	0.3		chr2:+212064031	1	2.12E+08	5	FALSE	
AGATAGAAATGAAAGACACTA	GTGAA	0.3		chr2:-141095548	-1	1.41E+08	5	FALSE	
TGATAAATGGGAATGACACCA	GAGAG	0.3		chr4:+146453372	1	1.46E+08	5	FALSE	

Mismatched nucleotides are colored red in sequence column,

*indicates off-target sites verified by PCR/Sanger sequencing using TZM-bl single cell clones

Supplementary Table 3. HIV-1 LTR1 and GagD target single clone off target analysis

OFF TARGET	Target sequence: ***LTR1*** PAM GCAGAACTACACACCAGGGCCAGGGAT<br>Predicted off target sequence:	Chromosome location/gene	Strand	Position	Score	Mismatches	Single cell clone	Number of sequences analyzed	Indels detected
1	ACAGCACTCCCCACCAGGGCTTGGGGG	Ch 16/TSC2	-	2082981	0.7	5	TOTAL	**32**	**0**
							CTRL1	6	0
							CTRL2	3	0
							ERAD1	3	0
							ERAD2	3	0
							ERAD3	3	0
							ERAD4	3	0
							ERAD5	5	0
							ERAD6	6	0
2	ACAAACCTACAGACCAGAGCCCAGGGT	Ch 11/TUB	-	8105357	0.4	5	TOTAL	**23**	**0**
							CTRL1	2	0
							CTRL2	3	0
							ERAD1	3	0
							ERAD2	3	0
							ERAD3	3	0
							ERAD4	3	0
							ERAD5	3	0
							ERAD6	3	0
3	TCTAAACTCCACACCAGGGCCATGAAG	Ch 12/SLC41A2	+	22915337	2.6	4	TOTAL	**19**	**0**
							CTRL1	2	0
							CTRL2	2	0
							ERAD1	3	0
							ERAD2	3	0
							ERAD3	3	0
							ERAD4	2	0
							ERAD5	3	0
							ERAD6	1	0
OFF TARGET	Target sequence: ***GagD*** **PAM** GGATAGATGTAAAAGACACCAAGGAAG<br>Predicted off target sequence:	Chromosome location/gene	Strand	Position	Score	Mismatches	Single cell clone	Number of sequences analyzed	Indels detected
1	CCAAAGATGAAAAAGACACCCGAGAAA	Ch 10/TACC2	+	122211347	0.6	5	TOTAL	**46**	**0**
							CTRL1	3	0
							CTRL2	3	0
							ERAD1	6	0
							ERAD2	3	0
							ERAD3	9	0
							ERAD4	6	0
							ERAD5	7	0
							ERAD6	9	0
2	TGAGACTTGTACAAGACACCACGGGGC	Ch 20/ADNP	-	50889247	0.5	5	TOTAL	**24**	**0**
							CTRL1	3	0
							CTRL2	3	0
							ERAD1	3	0
							ERAD2	3	0
							ERAD3	3	0
							ERAD4	3	0
							ERAD5	3	0
							ERAD6	3	0
3	AGAAAAATGTAAAAGACACCTTGGAAA	Ch 3/non gene	-	144746442	1.7	4	TOTAL	**21**	**0**
							CTRL1	3	0
							CTRL2	3	0
							ERAD1	3	0

							ERAD2	3	0
							ERAD3	2	0
							ERAD4	3	0
							ERAD5	1	0
							ERAD6	3	0

Target and predicted off-target sequences in human genome are highlighted green, PAM sequences in red, mismatched nucleotides in grey.

Supplementary Table 4. Number of somatic SNPs in different genomic regions

Sample	M4349	M4346	M4356
CDS	6590	6760	5895
Synonymous_SNP	3003	3053	2588
Missence_SNP	3372	3465	3050
Stopgain	70	86	128
Stoploss	6	6	7
Unknown	140	151	122
Intronic	414960	415812	382465
UTR3	8195	8197	7713
UTR5	1905	1947	1649
Splicing	23	27	30
ncRNA_exonic	3982	4073	3509
ncRNA_intronic	65272	65664	59525
ncRNA_splicing	14	14	13
Upstream	6962	7052	6063
Downstream	7822	7850	7005
Intergenic	664364	669294	602974
Total	1180363	1186971	1077074

Sample: sample name
CDS: the number of somatic SNPs in coding region
Synonymous_SNP: a single nucleotide change that does not cause an amino acid change
Missense_SNP: a single nucleotide change that causes an amino acid change
Stopgain: a nonsynonymous SNP that leads to the immediate creation of stop codon at the variant site
Stoploss: a nonsynonymous SNP that leads to the immediate elimination of stop codon at the variant site
Unknown: unknown function (due to various errors in the gene structure definition in the database file)
Intronic: the number of somatic SNPs in intronic region
UTR3: the number of somatic SNPs in 3'UTR region
UTR5: the number of somatic SNPs in 5'UTR region
Splicing: the number of somatic SNPs within 2-bp of a splicing junction
ncRNA_exonic: the number of somatic SNPs in exonic region of non-coding RNAs
ncRNA_intronic: the number of somatic SNPs in intronic region of non-coding RNAs
ncRNA_splicing: the number of somatic SNPs within 2-bp of a splicing junction of non-coding RNAs
Upstream: the number of somatic SNPs within 1kb away from the transcription start site
Downstream: the number of somatic SNPs within the 1kb away from the transcription termination site
Intergenic: the number of somatic SNPs in intergenic region
Total: the total number of somatic SNPs

Supplementary Table 5. Number of somatic InDels in different genomic regions

Sample	M4349	M4346	M4356
CDS	103	124	59
Frameshift_deletion	31	34	21
Frameshift_insertion	14	16	10
Nonframeshift_deletion	36	48	16
Nonframeshift_insertion	18	22	9
Stopgain	2	2	1
Stoploss	0	0	0
Unknown	2	2	2
Intronic	36969	39727	25080
UTR3	946	1003	640
UTR5	134	149	89
Splicing	5	5	3
ncRNA_exonic	285	314	190
ncRNA_intronic	5794	6247	3971
ncRNA_splicing	2	3	1
Upstream	694	771	417
Downstream	879	958	602
Intergenic	56749	61117	38406
Total	102588	110452	69477

Sample: sample name
CDS: the number of somatic InDels in coding region;
Frameshift_deletion: a deletion of one or more nucleotides that cause frameshift changes in protein coding sequence;
Frameshift_insertion: an insertion of one or more nucleotides that cause frameshift changes in protein coding sequence;
Nonframeshift_deletion: a deletion that does not cause frameshift changes;
Nonframeshift_insertion: an insertion that does not cause frameshift changes;
Stopgain: an insertion or a deletion that leads to the immediate creation of stop codon at the variant site;
Stoploss: an insertion or a deletion that leads to the immediate elimination of stop codon at the variant site;
Unknown: unknown function (due to various errors in the gene structure definition in the database file);
Intronic: the number of somatic InDels in intronic region; UTR3: the number of somatic InDels in 3’UTR region;
UTR5: the number of somatic InDels in 5’UTR region;
Splicing: the number of somatic InDels within 2-bp of a splicing junction;
ncRNA_exonic: the number of somatic InDels in exonic region of non-coding RNAs
ncRNA_intronic: the number of somatic InDels in intronic region of non-coding RNAs;
ncRNA splicing: the number of somatic InDels within 2-bp of a splicing junction of non-coding RNAs;
Upstream: the number of somatic InDels within 1kb away from transcription start site;
Downstream: the number of somatic InDels iwithin 1kb away from transcription termination site;
Intergenic: the number of somatic InDels in intergenic region;
Total: the total number of somatic InDels.

Supplementary Table 6. Viral rebound measure for three independent experiments

	HIV-1											
				LASER ART								
							CRISPR-Cas9					
Experiment	1st	2nd	3rd	1st	2nd	3rd	1st	2nd	3rd	1st	2nd	3rd
Mouse Numbers	6	6	7	10	2	5	7	6	10	6	NA	7
Viral Rebound*	6/6	4/4	7/7	10/10	2/2	5/5	5/7	3/6	6/10	6/6	NA	7/7

LASER ART was administered then stopped with consequent administration of AAV_9-CRISPR-Cas9. Plasma drug levels were monitored until they reached concentrations below the detection limit. These experiments were performed using HIV-1_{NL4-3} (one experiment) and HIV-1_{ADA} (two experiments) infections of humanized mice. Overall, sequential treatments with LASER ART and CRISPR-Cas9 showed absent viral rebound in up to or beyond a third of the tested animals. In these mice virus was eliminated from cell and tissue reservoirs. *Plasma viral RNA copies were > 10^4/ml.
